# Basal Vitamin D Status and Supplement Dose Are Primary Contributors to Maternal 25-Hydroxyvitamin D Response to Prenatal and Postpartum Cholecalciferol Supplementation

**DOI:** 10.1093/jn/nxab265

**Published:** 2021-07-24

**Authors:** Benjamin Levy, Karen M O'Callaghan, Huma Qamar, Abdullah Al Mahmud, Alison D Gernand, M Munirul Islam, Daniel E Roth

**Affiliations:** Centre for Global Child Health, Hospital for Sick Children, Toronto, Ontario, Canada; Centre for Global Child Health, Hospital for Sick Children, Toronto, Ontario, Canada; Centre for Global Child Health, Hospital for Sick Children, Toronto, Ontario, Canada; Nutrition and Clinical Services Division, International Centre for Diarrhoeal Disease Research, Bangladesh (icddr, b), Dhaka, Bangladesh; Department of Nutritional Sciences, The Pennsylvania State University, University Park, PA, USA; Nutrition and Clinical Services Division, International Centre for Diarrhoeal Disease Research, Bangladesh (icddr, b), Dhaka, Bangladesh; Centre for Global Child Health, Hospital for Sick Children, Toronto, Ontario, Canada; Department of Nutritional Sciences, Faculty of Medicine, University of Toronto, Toronto, Ontario, Canada; Department of Paediatrics, Hospital for Sick Children and University of Toronto, Toronto, Ontario, Canada

**Keywords:** 25-hydroxyvitamin D, dose–response, infancy, pregnancy, randomized controlled trial, vitamin D

## Abstract

**Background:**

Variability in the 25-hydroxyvitamin D [25(OH)D] response to prenatal and postpartum vitamin D supplementation is an important consideration for establishing vitamin D deficiency prevention regimens.

**Objectives:**

We aimed to examine interindividual variation in maternal and infant 25(OH)D following maternal vitamin D supplementation.

**Methods:**

In a randomized trial of maternal vitamin D supplementation (Maternal Vitamin D for Infant Growth Trial), healthy pregnant women (*n* = 1300) received a prenatal cholecalciferol (vitamin D-3) dose of 0, 4200, 16,800, or 28,000 IU/wk from 17 to 24 wk of gestation followed by placebo to 6 mo postpartum. A fifth group received 28,000 IU cholecalciferol/wk both prenatally and postpartum. In a subset of participants, associations of 25(OH)D with hypothesized explanatory factors were estimated in women at delivery (*n* = 655) and 6 mo postpartum (*n* = 566), and in their infants at birth (*n* = 502) and 6 mo of age (*n* = 215). Base models included initial 25(OH)D and supplemental vitamin D dose. Multivariable models were extended to include other individual characteristics and specimen-related factors. The model coefficient of determination (R^2^) was used to express the percentage of total variance explained.

**Results:**

Supplemental vitamin D intake and initial 25(OH)D accounted for the majority of variance in maternal 25(OH)D at delivery and postpartum (R^2^ = 70% and 79%, respectively). Additional characteristics, including BMI, contributed negligibly to remaining variance (<5% increase in R^2^). Variance in neonatal 25(OH)D was explained mostly by maternal delivery 25(OH)D and prenatal vitamin D intake (R^2^ = 82%). Variance in 25(OH)D in later infancy could only partly be explained by numerous biological, sociodemographic, and laboratory-related characteristics, including feeding practices (R^2^ = 43%).

**Conclusions:**

Presupplementation 25(OH)D and vitamin D supplemental dose are the major determinants of the response to maternal prenatal vitamin D intake. Vitamin D dosing regimens to prevent maternal and infant vitamin D deficiency should take into consideration the mean 25(OH)D concentration of the target population.

## Introduction

The high prevalence of vitamin D deficiency in pregnant women and newborns has been recognized as a global issue, with variability within and across WHO world regions (1). It is widely accepted that prenatal vitamin D supplementation presents a feasible strategy for the improvement of both maternal and neonatal vitamin D status (2), as reflected by increases in circulating 25-hydroxyvitamin D [25(OH)D] (3–5). However, efforts to recommend specific dosing regimens in the prenatal and postpartum period are hindered by a lack of pregnancy-specific Dietary Reference Values (DRVs) for vitamin D and limited data to establish DRVs beyond an adequate intake value in children <1 y of age (6). Establishing safe and effective supplemental intakes of vitamin D requires knowledge of the dose–response relation between 25(OH)D and vitamin D intake, with consideration of safety parameters (e.g., serum calcium) (6). Although the use of 25(OH)D as a standalone biomarker of vitamin D status has been disputed (7), in the absence of more robust metabolic end points and conclusive evidence for direct benefits of vitamin D on perinatal outcomes, prevention of low maternal and neonatal 25(OH)D remains the primary benchmark for establishing maternal vitamin D supplementation regimens during pregnancy (2).

Substantial heterogeneity in the 25(OH)D response to vitamin D intake has been observed in nonpregnant adults (8, 9), yet few dose-ranging trials have addressed the determinants of maternal circulating 25(OH)D in response to prenatal supplementation (10, 11), nor has this relation been extensively explored in maternal-infant dyads throughout lactation. Leveraging the placebo-controlled dose-ranging design of a previously reported vitamin D trial (12), we examined the biological, sociodemographic, and specimen/laboratory-related factors that contribute to interindividual variation in maternal and infant 25(OH)D following maternal vitamin D supplementation during pregnancy and the first 6 mo postpartum.

## Methods

### Study design and participant eligibility

This secondary analysis used data from participants of the Maternal Vitamin D for Infant Growth Trial of maternal prenatal and postpartum vitamin D supplementation in Dhaka, Bangladesh (12, 13). Briefly, generally healthy women (*n* = 1300), having an uncomplicated singleton pregnancy, were enrolled at 17–24 wk of gestation and randomly assigned to 1 of 5 equally sized trial arms, comprising a prenatal:postpartum regimen of 0:0 (placebo); 4200:0; 16,800:0; 28,000:0; or 28,000:28,000 IU cholecalciferol (vitamin D-3) per week until 26 wk postpartum. In addition to the intervention dose, all participants were provided with standard iron–folic acid supplements as per usual care, and calcium supplementation (500 mg/d calcium carbonate) to mitigate effects of low dietary calcium intakes. Ethical approval was obtained from the Research Ethics Committees of the Hospital for Sick Children in Toronto (REB1000039072) and the International Centre for Diarrhoeal Disease Research, Bangladesh (PR-13055). Written informed consent was provided by all women prior to commencing the trial. The trial was registered prospectively at clinicaltrials.gov (ID: NCT01924013).

To permit consideration of basal 25(OH)D in our modeling approach as a determinant of the vitamin D intake–25(OH)D response (14, 15), maternal-infant pairs enrolled in the parent trial were included in the present analysis if a maternal and/or infant 25(OH)D measurement was available for ≥2 consecutive study time points (i.e., maternal enrollment–delivery; maternal delivery–6 mo postpartum; maternal delivery–neonatal; or neonatal–6 mo of age).

### Intervention

Placebo and vitamin D-3 tablets were manufactured by the Toronto Institute of Pharmaceutical Technology. All tablets were identical in appearance, color, and taste. For each lot of tablets, manufacturer verification of the vitamin D content was confirmed by liquid chromatography–tandem mass spectrometry (LC-MS/MS) to a composition range within 10% of the labeled dose. Participant adherence was calculated by dividing the number of tablets consumed by the total number of assigned tablets. Average weekly supplemental vitamin D intake (referred to as “calculated dose per week” hereafter) was estimated from the measured tablet composition and individual participant adherence.

### Data collection

General health and sociodemographic information was collected at enrollment (17–24 wk of gestation) through interviewer-administered questionnaires. Asset index, as a proxy for socioeconomic status, was determined by ownership of household items, using principal components analysis (16). Habitual dietary calcium intakes in the prenatal and postpartum periods were estimated using a targeted, nonquantitative FFQ. Standard portion size estimates were obtained primarily from the Dietary Guidelines for Bangladesh (17), and supported by the Dietary Guidelines for Indians (18) and the Canadian Nutrient File (19). Calcium content was extracted from the Food Composition Table for Bangladesh (20) and the Helen Keller International Tables of Nutrient Composition of Bangladeshi Foods (21). Pre- and postnatal supplemental calcium was included in habitual intake estimates, using the percentage adherence to the vitamin D intervention as a proxy for compliance with the calcium co-intervention. Limited data on vitamin D intake were available from the FFQ at enrollment (22), which was expected to remain consistent throughout the observation period, and therefore not considered in the present study. Maternal, neonatal, and infant anthropometric indices were performed according to standardized protocols (23). BMI was calculated at enrollment based on a mid-pregnancy weight, because prepregnancy weight measurements were unavailable. Habitual BMI was derived from weight at 12 mo postpartum and used as a proxy for BMI in the nonpregnant, nonlactating state. Drained placental weight was measured following removal of the umbilical cord and membranes, to the nearest 0.5 g (iBALANCE i2500; My Weigh Canada). Nonfasting venous blood samples (including umbilical cord blood) were collected according to standard procedures and stored at ≤70°C until further analysis (12, 13). Infant feeding practices were assessed on a weekly basis to determine patterns of exclusive and combination breastfeeding, and the timing of introduction of complementary foods. At 6 mo of age, breastfeeding pattern was defined as exclusive/predominant breastfeeding (breast milk in addition to water, sugar water, honey, or other nonmilk, nonformula liquid), partial breastfeeding (breast milk with animal, powdered, or condensed milk, and solid or semisolid foods), or formula feeding.

### Laboratory analyses

Analysis of serum 25(OH)D was performed at the Analytical Facility for Bioactive Molecules (Hospital for Sick Children, Toronto), which participated in the Vitamin D External Quality Assessment Scheme (DEQAS; Charing Cross Hospital, London, UK) throughout the study period. Circulating 25(OH)D was measured in women (at enrollment, delivery, and 6 mo postpartum), umbilical cord blood, and infants (at 6 mo of age) by high-performance LC-MS/MS, using standardized methods that have been described in detail elsewhere (12). Chromatographic separation and quantification of 25(OH)D_3_, 3-epi-25-hydroxyvitamin D_3_, and 25-hydroxyvitamin D_2_ [25(OH)D_2_] was achieved. National Institute of Standards and Technology quality control materials (SRM 972a) and DEQAS standards (451, 452, 453, 454, 455) were used throughout the analysis. Mean inter- and intra-assay CVs for 25(OH)D_3_ were 7.0% and 4.9%, respectively. The lower limit of quantification (LLoQ) for 25(OH)D_3_ was 1.25 nmol/L (**Supplemental Table 1**). The LLoQ for 25(OH)D_2_ was 1.25 nmol/L; however, because 25(OH)D_2_ concentrations were undetectable or negligible in this cohort (12), only concentrations of 25(OH)D_3_ are reported in the present analysis, which excludes the C-3 epimer given the uncertainty regarding its biological relevance and activity (24). For simplicity, serum 25(OH)D concentrations are referred to as “25(OH)D” hereafter.

Additional maternal and infant metabolites, including circulating C-reactive protein (CRP), ferritin (maternal and infant), maternal serum folate and retinol, and infant serum creatinine were measured using commercially available kits (Supplemental Table 1). Inter- and intra-assay CVs for all analyses were <10%.

### Variables related to sample collection, storage, and laboratory procedures

To account for potential heterogeneity in 25(OH)D introduced through collection and handling of blood samples, we derived several sample-related variables: *1*) days since last dose was defined as the number of days between blood collection and the last administered intervention dose; *2*) time of day of blood collection was categorized as morning (00:00 to 11:59), afternoon (12:00 to 16:59), or evening (17:00 to 23:59); *3*) assay drift was represented by the number of months between completion of the first 25(OH)D assay and the assay concerning the 25(OH)D measurement of interest; and *4*), acknowledging the high stability of 25(OH)D (25), potential sample degradation during frozen storage was examined as the number of months between blood collection and 25(OH)D analysis. Because only freshly thawed serum samples were used for quantifying 25(OH)D, we did not need to account for variations introduced by multiple freeze-thaw samples (26). We calculated duration of prenatal supplementation as the number of weeks between randomization and delivery blood sample collection. Postpartum supplementation duration was calculated from the weeks between delivery and blood draw at ∼6 mo postdelivery.

### Statistical analysis

#### Data distributions and descriptive statistics

CRP and ferritin distributions were right-skewed and were therefore natural log-transformed to approximate normality. Pairwise correlations among variables were assessed using Pearson and Spearman correlation coefficients, and bivariate relations were examined using scatter plots with locally weighted regression. Biomarker concentrations below the LLoQ were assigned objective values equal to or half the LLoQ (Supplemental Table 1). Data were summarized as means ± SD or medians (25th percentile, 75th percentile) for continuous variables, and proportions and counts for categorical variables. Because inclusion in the present analysis required availability of 2 consecutive 25(OH)D measurements, participant characteristics were summarized separately for each time point of interest.

#### Sources of variation in the maternal and infant serum 25(OH)D response to maternal vitamin D supplementation

To identify factors associated with the 25(OH)D response to supplementation, we defined 4 intervals based on paired time points at which 25(OH)D was measured in maternal-infant pairs: *1*) maternal enrollment–delivery; *2*) maternal delivery–6 mo postpartum; *3*) maternal delivery–neonatal (umbilical cord), and *4*) neonatal–6 mo of age. Regression models were constructed with 25(OH)D as the (end point) outcome variable, measured at 4 corresponding (end of each interval) time points: *1*) maternal 25(OH)D at delivery; *2*) maternal 25(OH)D at 6 mo postpartum; *3*) neonatal 25(OH)D; and *4*) infant 25(OH)D at 6 mo of age. As a reflection of basal status, initial 25(OH)D was represented by the measurement at the preceding time point (i.e., start-of-interval of interest). For the maternal delivery and 6-mo postpartum end points, initial 25(OH)D was defined as enrollment and delivery 25(OH)D, respectively. For neonatal and infant 6-mo 25(OH)D, initial concentrations were defined as maternal delivery and neonatal 25(OH)D, respectively. We assumed that initial 25(OH)D accounted for nonsupplemental sources of vitamin D from cutaneous production and dietary intake, which was expected to be low relative to the supplemental doses provided (22).

For each interval, a base model included initial 25(OH)D and the calculated vitamin D dose per week as the only predictor variables. We then considered a range of other covariates that were hypothesized to be associated with 25(OH)D (**Supplemental Table 2**). For each interval and for each candidate factor, we first used a simple linear regression model (Model A) to estimate the unadjusted association of the hypothesized factor with 25(OH)D. We extended the model to adjust for initial 25(OH)D and calculated vitamin D dose per week (Model B), which enabled an assessment of the interindividual variation in end-of-interval 25(OH)D explained by the factor of interest, but which was not already explained in the base model. A parsimonious multivariable model (Model C) included initial 25(OH)D, calculated vitamin D dose per week, and any other covariate with a *P* value < 0.1 in its respective Model A and/or B, as well as selected variables that were a priori hypothesized to affect vitamin D status (season of blood draw, maternal BMI, and, for infants, breastfeeding pattern). Finally, for each interval, a multivariable model (Model D) consisted of all noncollinear covariates listed in Supplemental Table 2. Multivariable models C and D were used to determine the proportion of variance in end-of-interval 25(OH)D that could be explained by including a wide range of predictor variables. However, these models were not based on a specified causal framework and many of the factors were measured concurrently; therefore, the regression coefficients were cautiously interpreted and not assumed to indicate total or causal effects of each covariate. Pearson *r* ≥0.6 was used to define collinearity between pairs of continuous variables, and prompted consideration for removal from multivariable models. Gestational age at enrollment was tightly correlated with the duration of supplementation, as was maternal weight with BMI at each time point, and therefore only duration of supplementation and enrollment or habitual BMI remained in the final models.

**FIGURE 1:**
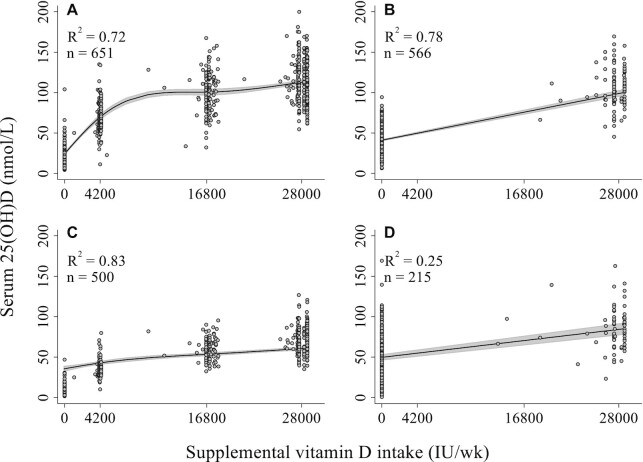
Scatter plots with fitted regression lines of the associations of calculated vitamin D dose per week with serum 25(OH)D at delivery. Variation in supplemental intake was captured by the verified vitamin D composition for each lot of tablets and individual participant adherence with the intervention. (A) Relation between attained maternal 25(OH)D concentrations at delivery and maternal prenatal supplemental vitamin D intake adjusting for maternal 25(OH)D concentrations at enrollment, modeled as a restricted cubic spline. (B) Relation between attained maternal 25(OH)D concentrations at 6 mo postpartum and maternal postpartum supplemental vitamin D intake adjusting for maternal delivery 25(OH)D concentrations, modeled as a linear function. (C) Relation between neonatal (umbilical cord) 25(OH)D concentrations and maternal prenatal supplemental vitamin D intake adjusting for maternal delivery 25(OH)D concentrations, modeled as a restricted cubic spline. (D) Relation between attained infant 25(OH)D concentrations at 6 mo of age and maternal postpartum supplemental vitamin D intake adjusting for umbilical cord 25(OH)D concentrations, modeled as a linear function. Owing to observed heteroskedasticity, robust SEs were estimated in all regression models for assessment of variation in 25(OH)D in response to vitamin D supplementation (Tables 2–5). 25(OH)D, 25-hydroxyvitamin D.

All covariates were modeled as fixed-effects. In an exploratory analysis, inclusion of the laboratory batch number as a random effect did not substantially increase the overall variance explained (data not shown); therefore, only results of fixed-effect models are reported. At each time point, statistical interactions were explored between vitamin D intake and each explanatory variable, including infant sex where applicable, to examine hypothesized effect modifiers of the vitamin D intake–25(OH)D relation. Interaction terms remained in Models B, C, and D if the Wald test for the interaction was statistically significant in Model B (*P* < 0.1). Irrespective of statistical significance, a decision was made a priori to include interaction terms for BMI and feeding practices in models concerning maternal delivery or neonatal 25(OH)D, and infant 6-mo 25(OH)D, respectively, based on known attributes of vitamin D metabolism [i.e., deposition of vitamin D in adipose tissue (27) and transfer of vitamin D to breast milk (28)]. For predictor variables that showed significant interactions with vitamin D intake, average marginal effects were computed and reported separately for each assigned intervention dose. Relations between maternal prenatal vitamin D intake and end-of-interval 25(OH)D, and between maternal delivery and neonatal 25(OH)D, were modeled using restricted cubic splines to account for observed nonlinearity of the 25(OH)D–vitamin D intake and fetomaternal 25(OH)D relations (**Figure 1**; **Supplemental Figure 1**, respectively). Knots were placed at values corresponding to the assigned doses for each trial arm (4200 IU/wk, 16,800 IU/wk, and 28,000 IU/wk). In women at delivery, a nonresponse to supplementation was nominally considered as failure to attain a 25(OH)D concentration ≥50% of the predicted mean 25(OH)D within each treatment group, based on a linear regression model including initial (enrollment) 25(OH)D, calculated vitamin D dose per week, and an interaction term between initial 25(OH)D and dose per week as predictor variables.

For all outcomes [i.e., attained 25(OH)D at the end of each interval], the goodness-of-fit for the base model, Model C, and Model D were compared using Akaike information criterion and partial F-tests, where *P* < 0.05 was defined as a significant improvement in model fit. R^2^ statistics from each regression model were used for comparison of total variance explained. In all models, robust SEs were employed to account for heteroskedasticity (29, 30). Analyses and figures were completed using Stata v15.1 (StataCorp) and Tableau v2020.3.

## Results

### Participant characteristics

Of 655 women with 25(OH)D measured at delivery, 471 (72%) had a complete dataset (i.e., all covariates of interest) for inclusion in multivariable models. Complete data were available for 541 of 566 (96%) women at 6 mo postpartum, 329 of 502 (66%) infants at birth, and 174 of 215 (81%) infants at 6 mo of age (**Supplemental Tables 3** and **4**). Participants were equally distributed across trial arms, and sociodemographic characteristics were similar among participants included at each time point (**Table 1**). Excluding placebo, and accounting for both lot variation and participant adherence, total maternal supplemental vitamin D intakes ranged from 1132 to 28672 IU/wk in the prenatal period, and between 1011 and 28672 IU/wk postpartum.

**FIGURE 3:**
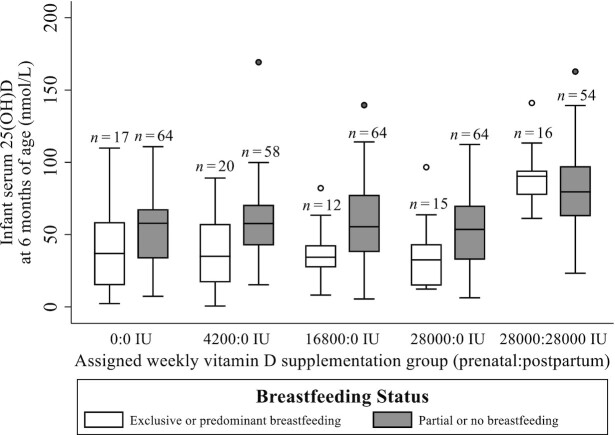
Comparison of infant 25(OH)D concentrations across treatment groups, and differentiated by breastfeeding pattern at 6 mo of age. Central line shows group median; lower and upper edges of the box denote 1st and 3rd quartile, respectively; lower and upper whiskers denote the lowest and highest values observed within 1.5 × IQR, respectively; outliers are represented by open (exclusive/predominant breastfeeding) and shaded (partial or no breastfeeding) dots. Placebo group is represented by 0:0 IU. *P* = 0.007 for interaction of breastfeeding pattern and vitamin D intake (as continuous variable) on infant 25(OH)D (multivariable Model D). 25(OH)D, 25-hydroxyvitamin D.

**TABLE 1:** Characteristics of participants included in multivariable models of attained serum 25-hydroxyvitamin D concentrations, by time point^1^

	Maternal delivery	Maternal 6 mo postpartum	Umbilical cord	Infant 6 mo of age
	(*n* = 471)	(*n* = 541)	(*n* = 329)	(*n* = 174)
Maternal characteristics				
Maternal age,^2^ y	22 (20, 26)	23 (20, 26)	22 (20, 25)	23 (20, 27)
Maternal height,^3^ cm	151.1 ± 5.7	151.0 ± 5.6	151.2 ± 5.7	151.1 ± 5.8
Maternal BMI at enrollment,^4^ kg/m^2^	23.0 (20.6, 26.3)	22.9 (20.6, 26.1)	22.9 (20.6, 26.4)	23.7 (21.0, 26.5)
<18.5, *n* (%)	39 (8.3)	42 (8.1)	28 (8.5)	9 (5.5)
≥18.5 to <25, *n* (%)	277 (59)	309 (59)	189 (57)	93 (57)
≥25.0 to <30, *n* (%)	123 (26)	142 (27)	90 (27)	56 (34)
≥30.0, *n* (%)	32 (6.8)	27 (5.2)	22 (6.7)	6 (3.7)
Maternal habitual BMI,^5^ kg/m^2^	23.4 (20.8, 26.5)	23.4 (20.7, 26.4)	23.5 (20.9, 26.8)	23.6 (20.6, 26.8)
<18.5, *n* (%)	47 (10)	55 (10)	35 (11)	17 (9.8)
≥18.5 to <25, *n* (%)	238 (52)	286 (53)	162 (51)	87 (50)
≥25.0 to <30, *n* (%)	130 (29)	156 (29)	89 (28)	54 (31)
≥30.0, *n* (%)	40 (8.8)	44 (8.1)	32 (10)	16 (9.2)
Gestational age at enrollment, wk	21 (19,22)	20 (19,22)	20 (19,22)	20 (19,22)
Parity,^6,7^*n* (%)				
Primiparous	225 (48)	249 (46)	157 (48)	70 (40)
Multiparous	246 (52)	292 (54)	172 (52)	104 (60)
Asset index quintile,^8^*n* (%)				
Q1	90 (19)	108 (20)	67 (20)	40 (23)
Q2	85 (18)	88 (16)	64 (19)	31 (18)
Q3	85 (18)	104 (19)	57 (17)	33 (19)
Q4	108 (23)	127 (23)	75 (23)	41 (24)
Q5	103 (22)	114 (21)	66 (20)	29 (17)
Maternal education level, *n* (%)				
No education	20 (4.2)	21 (3.9)	13 (4)	7 (4)
Primary; incomplete (grades 1–4)	93 (20)	107 (20)	62 (19)	36 (21)
Primary; complete (grade 5)	69 (15)	77 (14)	49 (15)	26 (15)
Secondary; incomplete (grades 6–9)	168 (36)	206 (38)	114 (35)	68 (39)
Secondary; complete or higher	121 (26)	130 (24)	91 (28)	37 (21)
Vitamin D intervention group,^9^*n* (%)				
0:0 IU/wk	92 (20)	105 (19)	59 (18)	38 (22)
4200:0 IU/wk	93 (20)	104 (19)	67 (20)	31 (18)
16,800:0 IU/wk	106 (23)	119 (22)	78 (24)	40 (23)
28,000:0 IU/wk	83 (18)	104 (19)	57 (17)	36 (21)
28,000:28,000 IU/wk	97 (21)	109 (20)	68 (21)	29 (17)
Enrollment serum 25(OH)D,^10^ nmol/L	27.1 ± 14.4	26.8 ± 14.2	27.3 ± 14.7	26.6 ± 15.1
Enrollment serum 25(OH)D <30 nmol/L,^10^*n* (%)	306 (65)	351 (65)	213 (65)	115 (66)
Prenatal calcium intake,^11^ mg/d	979 ± 267	992 ± 280	991 ± 245	1031 ± 314
Adherent to trial supplementation,^12^*n* (%)	455 (97)	528 (98)	319 (97)	172 (99)
Infant characteristics				
Sex				
Male	254 (54)	283 (52)	174 (53)	87 (50)
Female	217 (46)	258 (48)	155 (47)	87 (50)
Gestational age at birth, wk	39 (38, 40)	39 (38, 40)	39 (38, 40)	39 (38, 40)
Birth weight,^13^ g	2736 ± 341	2720 ± 341	2744 ± 346	2715 ± 364

1 Participants were considered eligible for inclusion in the present analysis provided a 25-hydroxyvitamin D measurement was available for 2 consecutive study time points (enrollment, delivery, umbilical cord, and 6 mo postdelivery), to account for effect modification by baseline (or preintervention) concentrations between each dosing interval. Inclusion in adjusted multivariable models (Models C and D) was dependent on data availability for corresponding covariates. 25(OH)D, 25-hydroxyvitamin D.

2 Data are presented as median (25th percentile, 75th percentile) (all such values).

3 Data are presented as mean ± SD (all such values).

4 *n* = 520 for analyses at 6 mo postpartum and *n* = 164 for infants at 6 mo of age.

5 BMI at 12 mo postpartum as a proxy for habitual BMI in the nonpregnant, nonlactating state; *n =* 455 for analyses at delivery and *n =* 318 for umbilical cord.

6 Data are presented as number (%) (all such values).

7 Defined based on the total number of previous live births, irrespective of previous miscarriage or abortions, and was inclusive of the current pregnancy. Hence, parity was categorized as primiparous (no previous live birth) or multiparous (≥1 live birth).

8 Determined by ownership of household items, using principal components analysis.

9 Denotes dose provided prenatally; dose provided postnatally.

10 *n =* 537 for analyses at 6 mo postpartum, *n =* 327 for umbilical cord, and *n =* 173 for infants at 6 mo of age.

11 Estimated by a targeted, nonquantitative FFQ, including calcium supplementation of 500 mg/d provided to all participants throughout the intervention period.

12 Defined a priori as consumption of ≥80% of scheduled tablets.

13 *n* = 414 for analyses among women at delivery and *n* = 472 among women at 6 mo postpartum.

### Comparison of models for estimating attained maternal and infant 25(OH)D following maternal vitamin D supplementation

The calculated vitamin D dose per week and initial 25(OH)D (base model) collectively explained a high proportion of total variance in maternal 25(OH)D at delivery and at 6 mo postpartum (70% and 79%, respectively) (Figure 1A; **Supplemental Table 5**). Addition of other covariates (Models C and D) yielded a statistically significant improvement in model fit (partial F-test: *P* < 0.01 for both), but with small increases in the proportion of variance explained (Supplemental Table 5). A similar trend was observed for neonatal 25(OH)D. Lower proportions of total variance in infant 25(OH)D at 6 mo of age were explained in the base model that included cord blood 25(OH)D and maternal postpartum calculated dose per week (24%). Considering goodness-of-fit and explanatory power collectively, the multivariable models (C or D) only provided meaningful explanatory benefit beyond calculated dose per week and initial 25(OH)D for infant concentrations at 6 mo of age (Supplemental Table 5).

### Factors associated with maternal 25(OH)D following prenatal and postpartum vitamin D supplementation

**FIGURE 2:**
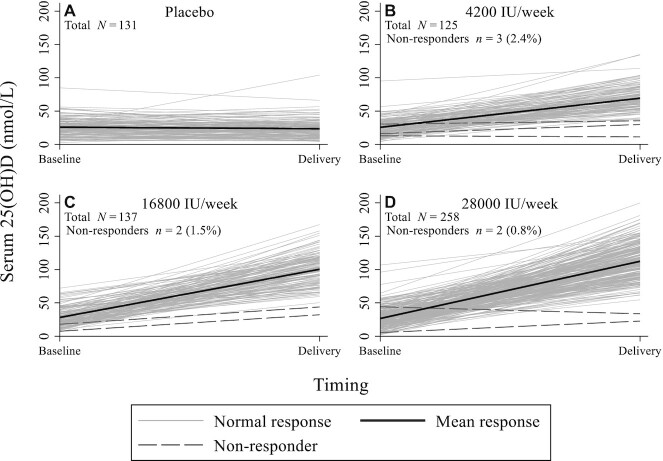
Response profiles of the vitamin D intake–25(OH)D relation in women at delivery. (A) Participants randomly assigned to placebo prenatally. (B) Participants randomly assigned to receive 4200 IU/wk vitamin D prenatally, (C) Participants randomly assigned to receive 16,800 IU/wk prenatally. (D) Participants randomly assigned to receive 28,000 IU/wk prenatally. A “nonresponse” to vitamin D supplementation was defined as a serum 25(OH)D concentration at delivery <50% of the predicted mean 25(OH)D within each treatment group, based on a linear regression model including enrollment 25(OH)D, calculated vitamin D dose per week, and an interaction term between enrollment 25(OH)D and dose per week as predictor variables. Robust SEs were estimated to account for heteroskedasticity. 25(OH)D, 25-hydroxyvitamin D.

There was a nonlinear relation between prenatal supplemental vitamin D intake and delivery 25(OH)D, with a major inflection point between intakes of 4200 IU/wk and 16,800 IU/wk (Figure 1A). Initial 25(OH)D did not significantly modify the effect of calculated vitamin D dose per week on maternal delivery 25(OH)D (*n* = 651; *P* = 0.31 for the interaction term). There was some evidence for attenuation of the response to supplementation at higher initial 25(OH)D, but effects were minor such that none of the interactions between vitamin D status classifications and calculated dose per week reached statistical significance (*P* = 0.71) (**Supplemental Figure 2**). Although there was considerable interindividual variation in response within each intervention group, few women (*n* = 7/520; 1.3%) achieved a delivery 25(OH)D <50% of the predicted group mean (**Figure 2**).

Postpartum vitamin D supplementation (28,000 IU/wk) had a potent effect on maternal 25(OH)D at 6 mo (Figure 1B). There was a significant interaction effect between maternal BMI and calculated dose per week on delivery and postpartum 25(OH)D, such that 25(OH)D was inversely associated with BMI in women receiving 28,000 IU/wk, but the effect of BMI was attenuated in the lower dose groups (**Tables 2** and **3**; **Supplemental Figure 3**). A similar interaction was found between maternal height and intervention dose in their effects on postpartum 25(OH)D (Table 3). Other maternal sociodemographic characteristics were not associated with maternal 25(OH)D in adjusted models (Tables 2 and 3). Of the biochemical markers measured at enrollment (CRP) and delivery (maternal folate, retinol, and ferritin), only folate was significantly (and positively) associated with attained 25(OH)D at delivery in multivariable models (Table 2). Maternal delivery 25(OH)D was higher from June to August relative to the winter months, but this association was only significant in Models B and C (Table 2; Supplemental Figure 3), and there was no significant seasonal effect on postpartum 25(OH)D (Table 3). The impact of sample collection and laboratory factors on 25(OH)D was inconsistent across the models and between delivery and postpartum time points; any significant effects were of small magnitude (Tables 2 and 3; Supplemental Figure 3).

**TABLE 2:** Unadjusted and multivariable-adjusted associations of maternal and specimen-related factors with the maternal serum 25-hydroxyvitamin D response to prenatal vitamin D-3 supplementation^1^

	Model A (unadjusted)		Model B [adjusted for initial 25(OH)D and calculated vitamin D dose/wk]		Model C [adjusted for initial 25(OH)D, calculated vitamin D dose/wk, and a limited set of additional covariates^2^]		Model D [adjusted for initial 25(OH)D, calculated vitamin D dose/wk, and all other listed covariates]	
	β (95% CI)^3^	*P*	β (95% CI)	*P*	β (95% CI)	*P*	β (95% CI)	*P*
Maternal enrollment serum 25(OH)D,^4^ nmol/L	0.50 (0.29, 0.71)	<0.001	—	—	0.39 (0.26, 0.52)	<0.001	0.36 (0.23, 0.50)	<0.001
Maternal age, y	0.02 (–0.72, 0.76)	0.95	–0.22 (–0.62, 0.19)	0.30	—	—	0.07 (–0.52, 0.66)	0.81
Maternal height, cm	–0.35 (–0.93, 0.23)	0.23	–0.20 (–0.51, 0.10)	0.19	—	—	–0.13 (–0.46, 0.21)	0.46
Maternal BMI at enrollment,^5^ kg/m^2^	–0.74 (–1.56, 0.09)	0.08	—	—	—	—	—	—
At 0 IU/wk	—	—	0.32 (–0.66, 1.30)	0.52	0.18 (–0.88, 1.23)	0.74	0.08 (–1.12, 1.28)	0.90
At 4200 IU/wk	—	—	–0.52 (–1.26, 0.23)	0.18	–0.23 (–1.11, 0.65)	0.61	–0.31 (–1.29, 0.68)	0.54
At 16,800 IU/wk	—	—	–0.46 (–1.50, 0.58)	0.39	–0.61 (–1.74, 0.52)	0.29	–0.69 (–1.91, 0.53)	0.27
At 28,000 IU/wk	—	—	–1.76 (–2.60, –0.92)	<0.001	–1.85 (–2.96, –0.74)	0.001	–1.73 (–2.83, –0.62)	0.002
Parity^5,6^								
Primiparous	ref.							
Multiparous	1.15 (–5.16, 7.47)	0.72	—	—	—	—	—	—
At 0 IU/wk	—	—	3.26 (–0.69, 7.21)	0.11	4.70 (–0.06, 9.46)	0.05	3.30 (–2.54, 9.13)	0.27
At 4200 IU/wk	—	—	–2.76 (–9.38, 3.86)	0.41	–3.65 (–11.78, 4.50)	0.38	–4.14 (–13.21, 4.96)	0.37
At 16,800 IU/wk	—	—	3.81 (–4.18, 11.80)	0.35	5.16 (–4.03, 14.35)	0.27	3.70 (–6.02, 13.43)	0.45
At 28,000 IU/wk	—	—	–4.92 (–11.15, 1.30)	0.12	–2.92 (–10.30, 4.46)	0.44	–2.73 (–10.53, 5.07)	0.49
Maternal education								
Secondary; complete or higher	ref.		ref.				ref.	
Secondary; incomplete (grades 6–9)	1.89 (–6.42, 10.20)	0.66	0.30 (–4.10, 4.70)	0.89	—	—	–0.39 (–5.85, 5.07)	0.89
Primary; complete (grade 5)	4.21 (–6.53, 14.96)	0.44	2.51 (–3.30, 8.31)	0.40	—	—	–1.53 (–8.30, 5.24)	0.66
Primary; incomplete (grades 1–4)	–1.54 (–11.17, 8.09)	0.75	0.33 (–4.41, 5.07)	0.89	—	—	–0.98 (–6.87, 4.91)	0.74
No education	–0.43 (–14.87, 14.01)	0.95	–7.54 (–17.44, 2.36)	0.14	—	—	–6.85 (–18.47, 4.76)	0.25
Asset index^7^	1.49 (–0.41, 3.40)	0.13	0.32 (–0.66, 1.30)	0.52	—	—	0.21 (–1.13, 1.55)	0.76
Paan use								
No	ref.		ref.				ref.	
Yes	–2.02 (–13.32, 9.27)	0.73	4.66 (–1.17, 10.49)	0.12	—	—	5.23 (–1.74, 12.21)	0.14
Prenatal calcium intake,^8^ g/d	6.68 (–5.04, 18.4)	0.26	–1.14 (–7.92, 5.64)	0.74	—	—	–3.07 (–11.42, 5.27)	0.44
Maternal enrollment plasma CRP, ln(μg/mL)	–0.68 (–3.40, 2.03)	0.62	–0.55 (–2.00, 0.90)	0.46	—	—	0.13 (–1.72, 1.98)	0.89
Maternal delivery serum retinol, μg/dL	0.03 (–0.25, 0.32)	0.81	—	—	—	—	—	—
At 0 IU/wk	—	—	0.02 (–0.22, 0.26)	0.88	—	—	0.10 (–0.17, 0.37)	0.45
At 4200 IU/wk	—	—	0.001 (–0.24, 0.24)	0.99	—	—	0.04 (–0.22, 0.30)	0.76
At 16,800 IU/wk	—	—	0.22 (–0.20, 0.63)	0.31	—	—	0.24 (–0.14, 0.63)	0.21
At 28,000 IU/wk	—	—	–0.39 (–0.68, –0.09)	0.010	—	—	–0.23 (–0.52, 0.05)	0.11
Maternal delivery serum folate, ng/mL	0.67 (–0.12, 1.45)	0.10	0.49 (0.05, 0.93)	0.029	0.66 (0.08, 1.24)	0.026	0.60 (–0.02, 1.22)	0.06
Maternal delivery serum ferritin, ln(ng/mL)	–3.66 (–7.57, 0.24)	0.07	—	—	—	—	—	—
At 0 IU/wk	—	—	–1.62 (–3.75, 0.51)	0.16	–1.99 (–5.59, 1.60)	0.28	–1.57 (–5.45, 2.32)	0.43
At 4200 IU/wk	—	—	–5.45 (–10.36, –0.54)	0.030	–4.33 (–10.49, 1.82)	0.17	–4.03 (–10.48, 2.43)	0.22
At 16,800 IU/wk	—	—	–6.69 (–12.31, –1.08)	0.019	–5.74 (–11.75, 0.26)	0.06	–6.80 (–12.42, –1.19)	0.018
At 28,000 IU/wk	—	—	0.50 (–3.06, 4.07)	0.78	0.66 (–3.43, 4.75)	0.75	1.38 (–2.93, 5.69)	0.53
Duration of supplementation, wk	0.95 (–0.36, 2.27)	0.16	0.27 (–0.48, 1.03)	0.48	—	—	–0.06 (–1.00, 0.88)	0.90
Season of blood collection								
Dec–Feb	ref.		ref.		ref.		ref.	
Mar–May	–2.81 (–12.97, 7.35)	0.59	4.04 (–1.57, 9.66)	0.16	0.99 (–5.27, 7.24)	0.76	–0.60 (–7.37, 6.17)	0.86
Jun–Aug	1.11 (–7.87, 10.08)	0.81	8.45 (3.63, 13.26)	0.001	8.84 (2.40, 15.28)	0.007	6.78 (–0.06, 13.62)	0.052
Sep–Nov	–2.30 (–10.18, 5.57)	0.57	1.59 (–2.65, 5.83)	0.46	0.80 (–3.78, 5.37)	0.73	–0.48 (–5.18, 4.21)	0.84
Time of day of blood collection^9^								
Morning	ref.		ref.				ref.	
Afternoon	5.32 (–2.31, 12.95)	0.17	2.53 (–1.62, 6.68)	0.23	—	—	–0.13 (–5.13, 4.86)	0.96
Evening	4.65 (–2.87, 12.18)	0.23	1.39 (–2.55, 5.33)	0.49	—	—	1.75 (–2.72, 6.22)	0.44
Days since last dose^10^	–1.32 (–2.38, –0.25)	0.016	–0.31 (–0.91, 0.29)	0.31	–0.32 (–0.96, 0.33)	0.34	–0.29 (–0.96, 0.38)	0.39
Time between blood collection and 25(OH)D analysis,^11^ mo	0.36 (–0.05, 0.77)	0.08	0.23 (–0.01, 0.47)	0.06	0.21 (–0.07, 0.50)	0.14	0.65 (0.24, 1.07)	0.002
Assay drift,^12^ mo	0.02 (–0.37, 0.40)	0.93	–0.08 (–0.30, 0.14)	0.47	—	—	–0.59 (–0.98, –0.21)	0.002

1 Model A was an unadjusted model with 25(OH)D at delivery as the outcome and the listed predictor as the exposure (1 individual model was created for each listed predictor). Model B additionally adjusted for maternal 25(OH)D at enrollment and calculated vitamin D dose per week (1 individual model was created for each listed predictor). Model C was a single parsimonious multivariable model that included enrollment 25(OH)D, calculated vitamin D dose per week, and any predictor variables with a *P* value <0.1 in Models A and/or B, alongside any variables that were a priori hypothesized to influence attained 25(OH)D concentrations. Model D adjusted for all listed hypothesized predictors in a single model. Robust SEs were estimated to account for heteroskedasticity. CRP, C-reactive protein; ref., reference; 25(OH)D, 25-hydroxyvitamin D.

2 Covariates adjusted for included: maternal BMI at enrollment, parity, maternal delivery serum folate, maternal delivery serum ferritin, season of blood collection, days since last dose, and time between blood collection and analysis.

3 β represents the difference in delivery 25(OH)D concentrations for every 1-unit increase in continuous variables or compared with the reference category for categorical variables. For selected predictor variables for which we examined interactions with vitamin D dose, associations are shown at specified supplemental vitamin D doses; in these instances, coefficients are predicted margins that represent the change in 25(OH)D for a 1-unit increase in the predictor (continuous variable) or difference compared with the reference category (categorical variable), at the specified vitamin D dose level.

4 Effect estimates of the association of enrollment 25(OH)D with delivery 25(OH)D are not shown for Model B because enrollment 25(OH)D was a covariate for all other predictors assessed. Hence, a different effect estimate was generated for 25(OH)D corresponding to each predictor variable investigated (Model B), rather than a single unique effect estimate for 25(OH)D.

5 Interaction with dose in adjusted and multivariate models.

6 Defined based on the total number of previous live births, irrespective of previous miscarriage or abortions, and was inclusive of the current pregnancy. Hence, parity was categorized as primiparous (no previous live birth) or multiparous (≥1 live birth).

7 Determined by ownership of household items, using principal components analysis.

8 Estimated by a targeted, nonquantitative FFQ.

9 Categorized as morning (00:00 to 11:59), afternoon (12:00 to 16:59), or evening (17:00 to 23:59).

10 Defined as the number of days between blood sample collection and the last administered vitamin D (or placebo) intervention dose, whereby the maximum number of days was truncated at 14.

11 Used to examine potential minor degradation during freezer storage.

12 Estimated by the number of months between completion of the first 25(OH)D assay and the assay concerning the 25(OH)D measurement of interest.

**TABLE 3:** Unadjusted and multivariable-adjusted associations of maternal and specimen-related factors with the maternal serum 25-hydroxyvitamin D response to postpartum vitamin D-3 supplementation^1^

	Model A (unadjusted)		Model B [adjusted for initial 25(OH)D and calculated vitamin D dose/wk]		Model C [adjusted for initial 25(OH)D, calculated vitamin D dose/wk, and a limited set of additional covariates^2^]		Model D [adjusted for initial 25(OH)D, calculated vitamin D dose/wk, and all other listed covariates]	
	β (95% CI)^3^	*P*	β (95% CI)	*P*	β (95% CI)	*P*	β (95% CI)	*P*
Maternal delivery serum 25(OH)D,^4^ nmol/L	0.45 (0.40, 0.50)	<0.001	—	—	0.26 (0.23, 0.28)	<0.001	0.25 (0.23, 0.28)	<0.001
Maternal age, y	0.07 (–0.55, 0.69)	0.82	–0.33 (–0.61, –0.04)	0.024	–0.12 (–0.42, 0.17)	0.41	–0.22 (–0.58, 0.14)	0.23
Maternal height,^5^ cm	0.16 (–0.27, 0.59)	0.46	—	—	—	—	—	—
At 0 IU/wk	—	—	0.01 (–0.18, 0.21)	0.90	0.03 (–0.16, 0.21)	0.79	0.04 (–0.15, 0.23)	0.66
At 28,000 IU/wk	—	—	–0.88 (–1.61, –0.15)	0.018	–0.90 (–1.56, –0.23)	0.008	–0.90 (–1.60, –0.20)	0.011
Maternal habitual BMI,^5,6^ kg/m^2^	–0.46 (–1.10, 0.19)	0.16	—	—	—	—	—	—
At 0 IU/wk	—	—	–0.29 (–0.54, –0.04)	0.023	–0.19 (–0.47, 0.09)	0.18	–0.24 (–0.52, 0.04)	0.10
At 28,000 IU/wk	—	—	–1.46 (–2.61, –0.31)	0.013	–1.27 (–2.38, –0.17)	0.025	–1.19 (–2.33, –0.06)	0.039
Parity^5,7^								
Primiparous	ref.						ref.	
Multiparous	3.29 (–1.89, 8.48)	0.21	—	—	—	—	—	—
At 0 IU/wk	—	—	0.85 (–1.45, 3.14)	0.47	—	—	1.50 (–1.52, 4.52)	0.33
At 28,000 IU/wk	—	—	–8.24 (–16.28, –0.21)	0.044	—	—	–1.64 (–10.27, 7.00)	0.71
Maternal education level								
Secondary; complete or higher	ref.		ref.				ref.	
Secondary; incomplete (grades 6–9)	4.66 (–1.97, 11.29)	0.17	2.03 (–0.99, 5.05)	0.19	—	—	1.84 (–1.18, 4.87)	0.23
Primary; complete (grade 5)	6.80 (–2.82, 16.43)	0.17	0.61 (–3.73, 4.95)	0.78	—	—	1.59 (–2.85, 6.03)	0.48
Primary; incomplete (grades 1–4)	2.40 (–5.24, 10.05)	0.54	–0.07 (–3.41, 3.26)	0.97	—	—	0.77 (–2.81, 4.36)	0.67
No education	0.30 (–10.37, 10.97)	0.96	–0.58 (–5.19, 4.04)	0.81	—	—	2.08 (–2.58, 6.74)	0.38
Asset index^8^	0.89 (–0.65, 2.44)	0.26	–0.14 (–0.87, 0.59)	0.71	—	—	0.13 (–0.65, 0.90)	0.75
Paan use								
No	ref.		ref.				ref.	
Yes	0.84 (–9.33, 11.02)	0.87	3.14 (–1.13, 7.42)	0.15	—	—	3.60 (–1.23, 8.43)	0.14
Postpartum calcium intake,^9^ g/d	0.00 (–11.72, 11.73)	0.99	–5.54 (–10.99, –0.09)	0.047	–0.25 (–5.98, 5.48)	0.93	–0.04, (–5.91, 5.84)	0.99
Breastfeeding pattern^10^								
Exclusive/predominant	ref.		ref.				ref.	
Partial	1.86 (–4.23, 7.96)	0.55	1.95 (–0.86, 4.75)	0.17	—	—	1.32 (–1.47, 4.11)	0.35
Duration of supplementation,^5^ wk	0.16 (–1.54, 1.86)	0.85	—	—	—	—	—	—
At 0 IU/wk	—	—	–1.27 (–1.96, –0.58)	<0.001	–8.14 (–17.69, 1.42)	0.09	–7.46 (–17.04, 2.12)	0.13
At 28,000 IU/wk	—	—	–25.03 (–46.47, –3.58)	0.022	–20.33 (–47.35, 6.69)	0.14	–20.96 (–47.93, 6.02)	0.13
Season of blood collection								
Dec–Feb	ref.		ref.		ref.		ref.	
Mar–May	1.23 (–5.35, 7.80)	0.71	–0.38 (–3.42, 2.66)	0.81	0.49 (–2.42, 3.41)	0.74	0.49 (–2.47, 3.46)	0.74
Jun–Aug	6.36 (–0.92, 13.63)	0.09	0.70 (–2.52, 3.93)	0.67	0.17 (–2.92, 3.27)	0.91	–0.03 (–3.18, 3.11)	0.98
Sep–Nov	–1.24 (–9.16, 6.68)	0.76	–2.11 (–6.12, 1.89)	0.30	–1.46 (–5.47, 2.54)	0.47	–1.64 (–5.73, 2.45)	0.43
Time of day of blood collection^11^								
Morning	ref.		ref.				ref.	
Afternoon	4.75 (–1.81, 11.30)	0.16	2.26 (–0.71, 5.23)	0.14	—	—	2.52 (–0.57, 5.60)	0.11
Days since last dose^5,12^	–0.24 (–1.01, 0.53)	0.54	—	—	—	—	—	—
At 0 IU/wk	—	—	0.97 (0.56, 1.38)	<0.001	0.72 (0.29, 1.16)	0.001	0.75 (0.31, 1.19)	0.001
At 28,000 IU/wk	—	—	–0.40 (–2.21, 1.41)	0.67	–0.06 (–1.69, 1.57)	0.94	–0.18 (–1.78, 1.42)	0.83
Time between blood collection and 25(OH)D analysis,^13^ mo	–1.72 (–3.27, –0.18)	0.029	–1.96 (–2.84, –1.08)	<0.001	–0.81 (–1.64, 0.02)	0.06	–0.90 (–1.75, –0.05)	0.037
Assay drift,^14^ mo	0.47 (–0.07, 1.02)	0.09	0.83 (0.59, 1.08)	<0.001	0.60 (0.32, 0.88)	<0.001	0.56 (0.28, 0.84)	<0.001

1 Model A was an unadjusted model with maternal 25(OH)D at 6 mo postpartum as the outcome and the listed predictor as the exposure (1 individual model was created for each listed predictor). Model B additionally adjusted for maternal 25(OH)D at delivery and calculated vitamin D dose per week (1 individual model was created for each listed predictor). Model C was a single parsimonious multivariable model that included delivery 25(OH)D, calculated vitamin D dose per week, and any predictor variables with a *P* value <0.1 in Models A and/or B, alongside any variables that were a priori hypothesized to influence attained 25(OH)D concentrations. Model D adjusted for all listed hypothesized predictors in a single model. Robust SEs were employed to account for heteroskedasticity. CRP, C-reactive protein; ref., reference; 25(OH)D, 25-hydroxyvitamin D.

2 Covariates adjusted for included: maternal age, maternal height, maternal habitual BMI, postpartum calcium intake, duration of supplementation, season of blood collection, days since last dose, time between blood collection and 25(OH)D analysis, and assay drift.

3 β represents the difference in maternal 6-mo postpartum 25(OH)D concentrations for every 1-unit increase in continuous variables or compared with the reference category for categorical variables. For selected predictor variables for which we examined interactions with vitamin D dose, associations are shown at specified supplemental vitamin D doses; in these instances, coefficients are predicted margins that represent the change in 25(OH)D for a 1-unit increase in the predictor (continuous variable) or difference compared with the reference category (categorical variable), at the specified vitamin D dose level.

4 Effect estimates of the association of delivery 25(OH)D with postpartum 25(OH)D are not shown for Model B because delivery 25(OH)D was a covariate for all other predictors assessed. Hence, a different effect estimate was generated for 25(OH)D corresponding to each predictor variable investigated (Model B), rather than a single unique effect estimate for 25(OH)D.

5 Interaction with dose in adjusted and multivariate models.

6 BMI at 12 mo postpartum as a proxy for habitual BMI in the nonpregnant, nonlactating state.

7 Defined based on the total number of previous live births, irrespective of previous miscarriage or abortions, and was inclusive of the current pregnancy. Hence, parity was categorized as primiparous (no previous live birth) or multiparous (≥1 live birth).

8 Determined by ownership of household items, using principal components analysis.

9 Estimated by means of a targeted, nonquantitative FFQ.

10 Defined as exclusive or predominant if breast milk was provided alone or in addition to water, sugar water, honey, or other nonmilk, nonformula liquid, and partial if breast milk was provided alongside animal, powdered, or condensed milk, and solid or semisolid foods.

11 Categorized as morning (00:00 to 11:59), afternoon (12:00 to 16:59), or evening (17:00 to 23:59); no blood sample collection occurred in the evening at this time point.

12 Defined as the number of days between blood sample collection and the last administered vitamin D (or placebo) intervention dose, whereby the maximum number of days was truncated at 14.

13 Used to examine potential minor degradation during freezer storage.

14 Estimated by the number of months between completion of the first 25(OH)D assay and the assay concerning the 25(OH)D measurement of interest.

### Factors associated with neonatal 25(OH)D following maternal prenatal vitamin D supplementation

Maternal 25(OH)D at delivery strongly correlated with neonatal concentrations (ρ = 0.87; Supplemental Figure 1). Minor variation in neonatal 25(OH)D was attributable to maternal prenatal vitamin D intake independent of maternal delivery 25(OH)D (Figure 1C). Maternal serum folate at delivery was negatively associated with neonatal 25(OH)D (**Table 4**), in contrast to its positive association with maternal delivery 25(OH)D. There was evidence for an interaction effect between maternal CRP and treatment group on neonatal 25(OH)D, but a clear dose-ranging effect was not observed. As seen for maternal 25(OH)D, seasonal associations and effects of sample/laboratory factors with neonatal 25(OH)D were inconsistent across models, and were minor relative to the overarching effect of maternal 25(OH)D (Table 4).

**TABLE 4:** Unadjusted and multivariable-adjusted associations of maternal, infant, and specimen-related factors with the neonatal (umbilical cord) serum 25-hydroxyvitamin D response to maternal prenatal vitamin D-3 supplementation^1^

	Model A (unadjusted)		Model B [adjusted for initial 25(OH)D and calculated vitamin D dose/wk]		Model C [adjusted for initial 25(OH)D, calculated vitamin D dose/wk, and a limited set of additional covariates^2^]		Model D [adjusted for initial 25(OH)D, calculated vitamin D dose/wk, and all other listed covariates]	
	β (95% CI)^3^	*P*	β (95% CI)	*P*	β (95% CI)	*P*	β (95% CI)	*P*
Maternal age, y	−0.02 (−0.59, 0.56)	0.96	−0.004 (−0.23, 0.22)	0.97	—	—	−0.04 (−0.42, 0.33)	0.82
Maternal height,^4^ cm	−0.27 (−0.67, 0.14)	0.20	—	—	—	—	—	—
At 0 IU/wk	—	—	0.10 (−0.02, 0.22)	0.09	0.04 (−0.17, 0.25)	0.69	0.08 (−0.17, 0.32)	0.53
At 4200 IU/wk	—	—	−0.24 (−0.59, 0.11)	0.18	−0.02 (−0.34, 0.29)	0.89	−0.03 (−0.36, 0.31)	0.88
At 16,800 IU/wk	—	—	−0.51 (−0.81, −0.22)	0.001	−0.29 (−0.65, 0.07)	0.11	−0.27 (−0.65, 0.11)	0.17
At 28,000 IU/wk	—	—	0.07 (−0.24, 0.37)	0.68	−0.11 (−0.49, 0.27)	0.58	−0.11 (−0.49, 0.27)	0.56
Maternal BMI at enrollment,^4^ kg/m^2^	−0.76 (−1.34, −0.18)	0.011	—	—	—	—	—	—
At 0 IU/wk	—	—	0.10 (−0.06, 0.25)	0.21	−0.06 (−0.41, 0.29)	0.73	−0.15 (−0.54, 0.25)	0.46
At 4200 IU/wk	—	—	−0.44 (−0.86, −0.03)	0.037	−0.36 (−0.87, 0.15)	0.17	−0.46 (−1.03, 0.11)	0.11
At 16,800 IU/wk	—	—	−0.54 (−0.99, −0.09)	0.020	−0.10 (−0.60, 0.41)	0.71	−0.16 (−0.66, 0.35)	0.55
At 28,000 IU/wk	—	—	−0.31 (−0.84, 0.22)	0.25	0.34 (−0.41, 1.09)	0.37	0.31 (−0.44, 1.05)	0.42
Parity^5^								
Primiparous	ref.		ref.				ref.	
Multiparous	1.34 (−3.25, 5.92)	0.57	0.46 (−1.39, 2.31)	0.62	—	—	2.55 (−0.70, 5.80)	0.12
Maternal education								
Secondary; complete or higher	ref.		ref.				ref.	
Secondary; incomplete (grades 6–9)	3.16 (−2.84, 9.15)	0.30	1.04 (−1.43, 3.51)	0.41	—	—	−1.24 (−4.50, 2.02)	0.45
Primary; complete (grade 5)	3.33 (−4.37, 11.03)	0.40	1.04 (−2.12, 4.20)	0.52	—	—	−1.56 (−5.62, 2.51)	0.45
Primary; incomplete (grades 1–4)	−1.15 (−7.88, 5.57)	0.74	−0.09 (−2.78, 2.61)	0.95	—	—	−3.31 (−6.81, 0.20)	0.06
No education	5.02 (−6.20, 16.24)	0.38	1.41 (−4.38, 7.20)	0.63	—	—	−0.28 (−7.71, 7.16)	0.94
Asset index^6^	0.58 (−0.80, 1.96)	0.41	−0.34 (−0.89, 0.21)	0.23	—	—	−0.49 (−1.21, 0.24)	0.19
Maternal prenatal calcium intake,^7^ g/d	7.22 (−1.24, 15.68)	0.09	2.13 (−1.35, 5.60)	0.23	2.35 (−2.28, 6.99)	0.32	3.19 (−1.78, 8.16)	0.21
Maternal enrollment plasma CRP,^4^ ln(μg/mL)	−1.29 (−3.24, 0.67)	0.20	—	—	—	—	——	—
At 0 IU/wk	—	—	0.33 (−0.20, 0.87)	0.22	0.93 (−0.31, 2.17)	0.14	1.26 (−0.19, 2.71)	0.09
At 4200 IU/wk	—	—	−0.05 (−1.25, 1.15)	0.94	0.69 (−0.73, 2.11)	0.34	0.86 (−0.80, 2.51)	0.31
At 16,800 IU/wk	—	—	−2.22 (−3.92, −0.51)	0.011	−2.46 (−4.24, −0.67)	0.007	−2.59 (−4.40, −0.78)	0.005
At 28,000 IU/wk	—	—	−1.00 (−2.50, 0.51)	0.19	−1.91 (−4.11, 0.30)	0.09	−2.02 (−4.34, 0.31)	0.09
Maternal delivery serum retinol, μg/dL	0.07 (−0.12, 0.27)	0.46	0.03 (−0.05, 0.12)	0.46	—	—	0.02 (−0.09, 0.12)	0.72
Maternal delivery serum folate, ng/mL	−0.14 (−0.64, 0.36)	0.58	−0.24 (−0.44, −0.04)	0.017	−0.37 (−0.66, −0.08)	0.011	−0.31 (−0.65, 0.03)	0.07
Maternal delivery serum ferritin, ln(ng/mL)	−2.22 (−5.12, 0.67)	0.13	−0.29 (−1.44, 0.87)	0.63	—	—	−0.41 (−2.04, 1.23)	0.63
Gestational age at birth, wk	0.56 (−1.15, 2.27)	0.52	−0.03 (−0.74, 0.69)	0.94	—	—	0.76 (−0.43, 1.95)	0.21
Birth weight, g	−0.004 (−0.01, 0.003)	0.25	−0.002 (−0.005, 0.001)	0.13	—	—	−0.003 (−0.01, 0.003)	0.30
Placental weight, g	0.003 (−0.03, 0.03)	0.84	−0.002 (−0.02, 0.01)	0.80	—	—	0.01 (−0.01, 0.03)	0.39
Duration of supplementation, wk	0.35 (−0.66, 1.37)	0.49	−0.41 (−0.86, 0.03)	0.07	−0.42 (−0.95, 0.11)	0.124	−0.49 (−1.17, 0.18)	0.15
Season of blood collection								
Dec–Feb	ref.		ref.		ref.		ref.	
Mar–May	−5.80 (−12.58, 0.98)	0.09	−3.16 (−6.14, −0.18)	0.037	−2.21 (−5.45, 1.04)	0.18	−2.11 (−5.70, 1.49)	0.25
Jun–Aug	−3.93 (−10.13, 2.26)	0.21	−1.49 (−4.00, 1.02)	0.24	−0.31 (−3.98, 3.35)	0.87	−0.27 (−3.89, 3.36)	0.89
Sep–Nov	−4.58 (−10.58, 1.42)	0.14	−2.05 (−4.63, 0.53)	0.12	−3.57 (−6.60, −0.55)	0.021	−3.39 (−6.46, −0.31)	0.031
Time of day of blood collection^8^								
Morning	ref.		ref.		ref.		ref.	
Afternoon	−0.32 (−6.13, 5.48)	0.91	2.60 (0.27, 4.93)	0.029	1.27 (−1.83, 4.38)	0.42	1.05 (−2.12, 4.21)	0.52
Evening	6.92 (1.70, 12.15)	0.009	2.15 (−0.07, 4.38)	0.06	1.71 (−0.96, 4.39)	0.21	1.51 (−1.23, 4.24)	0.28
Days since last dose^9^	−0.50 (−1.29, 0.29)	0.21	0.18 (−0.16, 0.52)	0.30	—	—	0.30 (−0.11, 0.70)	0.15
Time between blood collection and 25(OH)D analysis,^10^ mo	0.44 (0.10, 0.78)	0.012	−0.01 (−0.20, 0.19)	0.95	−0.27 (−0.54, −0.001)	0.049	−0.28 (−0.56, −0.01)	0.042
Assay drift,^11^ mo	0.73 (0.28, 1.17)	0.001	0.24 (0.02, 0.46)	0.036	0.49 (0.17, 0.81)	0.003	0.50 (0.16, 0.84)	0.004

1 Model A was an unadjusted model with neonatal (umbilical cord) 25(OH)D as the outcome and the listed predictor as the exposure (1 individual model was created for each listed predictor). Model B additionally adjusted for maternal 25(OH)D at delivery and calculated vitamin D dose per week (1 individual model was created for each listed predictor). Model C was a single parsimonious multivariable model that included delivery 25(OH)D, calculated vitamin D dose per week, and any predictor variables with a *P* value <0.1 in Models A and/or B, alongside any variables that were a priori hypothesized to influence attained 25(OH)D concentrations. Model D adjusted for all listed hypothesized predictors in a single model. Robust SEs were estimated to account for heteroskedasticity. CRP, C-reactive protein; ref., reference; 25(OH)D, 25-hydroxyvitamin D.

2 Covariates adjusted for included: maternal height, maternal BMI at enrollment, maternal prenatal calcium intake, maternal enrollment plasma CRP, maternal delivery serum folate, duration of supplementation, season of blood collection, time of day of blood collection, time between blood collection and 25(OH)D analysis, and assay drift.

3 β represents the difference in venous cord 25(OH)D concentrations for every 1-unit increase in continuous variables or compared with the reference category for categorical variables. Estimates for the association between maternal delivery 25(OH)D with cord 25(OH)D were modeled using restricted cubic splines and are therefore not presented in multivariable models in this table (Models A–D, inclusive). For selected predictor variables for which we examined interactions with vitamin D dose, associations are shown at specified supplemental vitamin D doses; in these instances, coefficients are predicted margins that represent the change in 25(OH)D for a 1-unit increase in the predictor (continuous variable) or difference compared with the reference category (categorical variable), at the specified vitamin D dose level.

5 Defined based on the total number of previous live births, irrespective of previous miscarriage or abortions, and was inclusive of the current pregnancy. Hence, parity was categorized as primiparous (no previous live birth) or multiparous (≥1 live birth).

6 Determined by ownership of household items, using principal components analysis.

7 Estimated by a targeted, nonquantitative FFQ.

8 Categorized as morning (00:00 to 11:59), afternoon (12:00 to 16:59), or evening (17:00 to 23:59).

9 Defined as the number of days between blood sample collection and the last administered vitamin D (or placebo) intervention dose, whereby the maximum number of days was truncated at 14.

10 Used to examine potential minor degradation during freezer storage.

11 Estimated by the number of months between completion of the first 25(OH)D assay and the assay concerning the 25(OH)D measurement of interest.

### Factors associated with infant 25(OH)D at 6 mo of age following maternal vitamin D supplementation

Maternal postpartum vitamin D supplementation significantly increased infant serum 25(OH)D at 6 mo of age (Figure 1D). Of the maternal characteristics considered, only education level was associated with infant 25(OH)D, but a clear trend was not evident (**Table 5**). In the absence of maternal postpartum vitamin D supplementation, exclusive or predominant breastfeeding was associated with significantly lower infant 25(OH)D compared with partial breastfeeding or formula feeding (**Figure 3**; Table 5). However, the magnitude of this difference was attenuated by maternal vitamin D supplementation throughout lactation (Table 5), resulting in similar 25(OH)D concentrations in infants irrespective of mode of feeding (Figure 3). As observed for the other intervals, associations of sample/laboratory factors with infant 25(OH)D were inconsistent and difficult to disentangle from other factors in multivariable models (Table 5).

**TABLE 5:** Unadjusted and multivariable-adjusted associations of maternal, infant, and specimen-related factors with the infantile serum 25-hydroxyvitamin D response to maternal postpartum vitamin D-3 supplementation^1^

	Model A (unadjusted)		Model B [adjusted for initial 25(OH)D and calculated vitamin D dose/wk]		Model C [adjusted for initial 25(OH)D, calculated vitamin D dose/wk, and a limited set of additional covariates^2^]		Model D [adjusted for initial 25(OH)D, calculated vitamin D dose/wk, and all other listed covariates]	
	β (95% CI)^3^	*P*	β (95% CI)	*P*	β (95% CI)	*P*	β (95% CI)	*P*
Umbilical cord 25(OH)D,^4^ nmol/L	0.22 (0.07, 0.37)	0.005	—	—	0.05 (−0.10, 0.20)	0.52	0.04 (−0.11, 0.19)	0.57
Maternal age, y	−0.48 (−1.32, 0.37)	0.26	−0.65 (−1.37, 0.07)	0.08	−0.15 (−0.92, 0.63)	0.71	−0.63 (−1.62, 0.35)	0.21
Maternal height, cm	−0.29 (−0.86, 0.28)	0.32	−0.45 (−0.93, 0.03)	0.06	−0.21 (−0.77, 0.35)	0.46	−0.26 (−0.82, 0.29)	0.35
Maternal habitual BMI,^5^ kg/m^2^	0.07 (−0.85, 1.00)	0.88	0.12 (−0.72, 0.96)	0.78	—	—	1.10 (0.11, 2.08)	0.029
Parity^6^								
Primiparous	ref.		ref.				ref.	
Multiparous	0.65 (−6.66, 7.97)	0.86	−0.98 (−7.36, 5.40)	0.76	—	—	2.31 (−6.50, 11.11)	0.60
Maternal education level								
Secondary; complete or higher	ref.		ref.		ref.		ref.	
Secondary; incomplete (grades 6–9)	11.54 (1.47, 21.60)	0.024	11.29 (2.87, 19.71)	0.008	11.95 (2.69, 21.20)	0.011	13.48 (4.08, 22.89)	0.005
Primary; complete (grade 5)	−1.42 (−13.55, 10.70)	0.82	−0.62 (−10.63, 9.40)	0.90	−1.86 (−12.77, 9.06)	0.74	0.58 (−11.43, 12.59)	0.92
Primary; incomplete (grades 1–4)	6.17 (−5.85, 18.18)	0.31	3.93 (−5.72, 13.58)	0.42	5.24 (−5.79, 16.28)	0.35	6.16 (−4.64, 16.96)	0.26
No education	−0.74 (−20.62, 19.14)	0.94	0.84 (−15.12, 16.80)	0.92	4.11 (−14.59, 22.81)	0.66	6.59 (−12.16, 25.33)	0.49
Asset index^7^	0.001 (−2.05, 2.05)	0.99	0.33 (−1.54, 2.20)	0.73	—	—	1.47 (−0.80, 3.74)	0.20
Birth weight, g	−0.01 (−0.02, 0.01)	0.33	−0.01 (−0.02, 0.005)	0.28	—	—	−0.01 (−0.02, −0.002)	0.021
Gestational age at birth, wk	0.01 (−2.67, 2.68)	0.99	−0.66 (−2.94, 1.62)	0.57	—	—	−0.80 (−3.50, 1.90)	0.56
Sex								
Male	ref.		ref.		ref.		ref.	
Female	−6.27 (−13.62, 1.08)	0.09	−3.94 (−10.42, 2.55)	0.23	−3.63 (−10.67, 3.41)	0.31	−4.32 (−12.00, 3.36)	0.27
Breastfeeding pattern^8,9^								
Exclusive/dominant	ref.		ref.		ref.		ref.	
Partial	12.97 (3.31, 22.63)	0.009	—	—	—	—	—	—
Partial at 0 IU/wk	—	—	18.98 (10.65, 27.31)	<0.001	15.82 (6.40, 25.25)	0.001	13.84 (4.51, 23.16)	0.004
Partial at 28,000 IU/wk	—	—	−0.77 (−12.43, 10.89)	0.90	−3.69 (−17.16, 9.77)	0.59	−6.91 (−19.17, 5.36)	0.27
Infant 6-mo plasma CRP, ln(μg/mL)	−0.12 (−3.89, 3.65)	0.95	−0.56 (−4.03, 2.91)	0.75	—	—	—	—
Infant 6-mo serum creatinine, ng/mL	0.53 (−0.67, 1.72)	0.38	0.37 (−0.70, 1.45)	0.50	—	—	0.51 (−0.64, 1.66)	0.38
Infant 6-mo serum ferritin, ln(ng/mL)	3.81 (−0.40, 8.01)	0.08	3.26 (−0.65, 7.16)	0.10	2.19 (−1.66, 6.04)	0.26	3.22 (−0.92, 7.37)	0.12
Season of blood collection								
Dec–Feb	ref.		ref.		ref.		ref.	
Mar–May	−2.37 (−12.11, 7.38)	0.63	−1.58 (−9.87, 6.71)	0.71	−2.57 (−11.33, 6.18)	0.56	−1.69 (−10.63, 7.24)	0.71
Jun–Aug	1.20 (−9.54, 11.94)	0.83	2.36 (−6.74, 11.47)	0.61	2.53 (−6.79, 11.85)	0.59	3.05 (−5.74, 11.85)	0.49
Sep–Nov	4.69 (−9.86, 19.23)	0.53	5.21 (−9.08, 19.51)	0.47	4.00 (−10.34, 18.33)	0.58	3.90 (−10.72, 18.52)	0.60
Time of day of blood collection^10^								
Morning	ref.		ref.				ref.	
Afternoon	−1.09 (−11.03, 8.86)	0.83	−0.47 (−9.04, 8.10)	0.91	—	—	−1.38 (−10.21, 7.45)	0.76
Days since last dose^11^	0.39 (−0.93, 1.72)	0.56	1.16 (−0.04, 2.36)	0.059	1.31 (0.08, 2.55)	0.036	1.28 (0.08, 2.49)	0.036
Time between blood collection and 25(OH)D analysis,^12^ mo	0.50 (−0.85, 1.86)	0.47	0.54 (−0.67, 1.75)	0.38	−0.54 (−1.92, 0.84)	0.44	−0.98 (−2.19, 0.23)	0.11
Assay drift,^13^ mo	0.66 (−0.03, 1.34)	0.06	0.66 (0.08, 1.25)	0.027	0.53 (−0.25, 1.30)	0.18	0.65 (−0.11, 1.41)	0.09

1 Model A was an unadjusted model with infant serum 25(OH)D at 6 mo as the outcome and the listed predictor as the exposure (1 individual model was created for each listed predictor). Model B additionally adjusted for maternal 25(OH)D at delivery and calculated vitamin D dose per week (1 individual model was created for each listed predictor). Model C was a single parsimonious multivariable model that included umbilical cord 25(OH)D, calculated vitamin D dose per week, and any predictor variables with a *P* value <0.1 in Models A and/or B, alongside any variables that were a priori hypothesized to influence attained 25(OH)D concentrations. Model D adjusted for all listed hypothesized predictors in a single model. Robust SEs were estimated to account for heteroskedasticity. CRP, C-reactive protein; ref., reference; 25(OH)D, 25-hydroxyvitamin D.

2 Covariates adjusted for included: maternal age, maternal height, maternal education level, infant sex, breastfeeding pattern, infant 6-mo serum ferritin, season of blood collection, days since last dose, time between blood collection and analysis, and assay drift.

3 β represents the difference in infant 6-mo 25(OH)D concentrations for every 1-unit increase in continuous variables or compared with to the reference category for categorical variables. For selected predictor variables for which we examined interactions with vitamin D dose, associations are shown at specified supplemental vitamin D doses; in these instances, coefficients are predicted margins that represent the change in 25(OH)D for a 1-unit increase in the predictor (continuous variable) or difference compared with the reference category (categorical variable), at the specified vitamin D dose level.

4 Effect estimates of the association of umbilical cord 25(OH)D with infant 6-mo 25(OH)D are not shown for Model B because umbilical cord 25(OH)D was a covariate for all other predictors assessed. Hence, a different effect estimate was generated for 25(OH)D corresponding to each predictor variable investigated (Model B), rather than a single unique effect estimate for 25(OH)D.

5 BMI at 12 mo postpartum as a proxy for habitual BMI in the nonpregnant, nonlactating state.

6 Defined based on the total number of previous live births, irrespective of previous miscarriage or abortions, and was inclusive of the current pregnancy. Hence, parity was categorized as primiparous (no previous live birth) or multiparous (≥1 live birth).

7 Determined by claimed ownership of household items, using principal components analysis.

8 Defined as exclusive or predominant if breast milk was provided alone or in addition to water, sugar water, honey, or other nonmilk, nonformula liquid, and partial if breast milk was provided alongside animal, powdered, or condensed milk, and solid or semisolid foods.

9 Interaction with dose in adjusted and multivariate models.

10 Categorized as morning (00:00 to 11:59), afternoon (12:00 to 16:59), or evening (17:00 to 23:59); no blood sample collection occurred in the evening at this time point.

11 Defined as the number of days between blood sample collection and the last administered vitamin D intervention (or placebo) dose, whereby the maximum number of days was truncated at 14.

12 Used to examine potential minor degradation during freezer storage.

13 Estimated by the number of months between completion of the first 25(OH)D assay and the assay concerning the 25(OH)D measurement of interest.

## Discussion

Determination of safe and effective strategies for prevention of micronutrient deficiencies is reliant on an understanding of interindividual variation in the response to supplementation and dietary intake (31). Despite the known alterations in vitamin D metabolism that occur during pregnancy (32, 33), few studies to date have explored the sources of variability in the 25(OH)D response to prenatal and postpartum vitamin D supplementation (10, 11). Here, we found that a substantial majority of heterogeneity in the maternal 25(OH)D dose–response during pregnancy and lactation was explained by just 2 factors—vitamin D supplement intake and initial circulating 25(OH)D. Other biological and sociodemographic characteristics explained little additional variance in 25(OH)D. Because the determinants of habitual vitamin D status were expected to be reflected in the initial 25(OH)D concentration, the multivariable modeling approach in this study was aimed at identifying factors that explained differences in attained 25(OH)D beyond those attributable to initial vitamin D status and intake. In agreement with previous findings, our results confirm neonatal 25(OH)D strongly reflects maternal vitamin D status in late gestation (34–36) and highlights the important contribution of early feeding practices to infant vitamin D status in the first 6 mo of life.

Despite recent efforts to bridge knowledge gaps surrounding dietary vitamin D requirements, interindividual variability in the dose–response relation of 25(OH)D to vitamin D intake remains a relatively understudied consideration in the estimation of requirements (37). Between-individual variability is expected and can be minimized to some extent in prediction models by adjusting for known physiological covariates, including age and basal vitamin D status (38, 39). Beyond the recognized effect of supplementation adherence, our analysis showed very few women fail to respond to supplementation with vitamin D; only ∼1% attained a 25(OH)D concentration below the expected range (<50% of predicted) at delivery. Even if these few individuals were genuine “nonresponders” [rather than being explained by errors in 25(OH)D measurements], this finding nonetheless reinforces the concept that, in a generally healthy population with known basal 25(OH)D, supplementation yields a predictable rise in circulating 25(OH)D.

The present analysis extends the previous work by Moon and colleagues (10, 40), who also reported determinants of 25(OH)D in response to prenatal vitamin D supplementation in the context of a placebo-controlled trial. Despite differences in study design and population demographics, our findings, together with those of the Maternal Vitamin D Osteoporosis Study (MAVIDOS), collectively highlight the importance of basal 25(OH)D as a determinant of attained 25(OH)D following antenatal supplementation. The rise in maternal 25(OH)D following supplementation occurred in parallel with initial 25(OH)D, such that final achieved 25(OH)D concentrations in women receiving the same dose were greatest in women with higher 25(OH)D prior to randomization. In contrast to MAVIDOS, but in line with our earlier findings of pooled trial data (41), we did not observe a greater achieved 25(OH)D in response to supplementation in women with moderate levels of deficiency [25(OH)D: ≥15 to <30 nmol/L] at enrollment compared with women who were relatively vitamin D replete (≥30 nmol/L). The present findings were likely due to the high prevalence of deficiency in our study population prior to intervention, in addition to the comparatively high doses of vitamin D provided relative to habitual intakes (22).

Consistent with previous intervention trials, including pregnant (4), pediatric (42), and adolescent (43) populations, the present dose-ranging analysis shows the slope reflecting the rise in 25(OH)D per unit increase in vitamin D intake attenuates with increasing dose, and there is a near plateauing of 25(OH)D beyond prenatal intakes of 16,800 IU/wk. At the highest assigned vitamin D intervention dose only (28,000 IU/wk), the strength of the dose–response relation was inversely associated with maternal BMI. Due to volumetric dilution across a larger tissue mass (44, 45), greater adiposity or body mass is proposed to lead to an overall increased requirement for vitamin D to achieve a given 25(OH)D, which could be an important consideration when developing dietary guidelines (46, 47). Similar associations with BMI were observed in 2 previous trials of comparatively lower doses of vitamin D. Moon et al. (10) reported an inverse association between first-trimester BMI and maternal 25(OH)D following vitamin D supplementation (1000 IU/d), and Alhomaid et al. (48) found that early-pregnancy obesity was associated with an attenuated 25(OH)D response to prenatal vitamin D supplementation (800 IU/d). Although we found the association with BMI to be weaker at vitamin D doses equivalent to 2400 IU/d or lower, this might have been attributable to the lower proportion of women with high BMI (i.e., >30 kg/m^2^) compared with the previous trial populations.

A substantial proportion of variance in 25(OH)D remained unexplained for infants at 6 mo of age. As dietary diversity increases with the introduction of complementary foods, and sun exposure behavior influences cutaneous vitamin D synthesis, the difficulty of capturing direct sources of variation in 25(OH)D increases with advancing age. We found that maternal postpartum supplementation attenuated the difference in 25(OH)D between breastfed and formula-fed infants, results that corroborate previous findings that high-dose maternal vitamin D supplementation can increase breast milk antirachitic activity, in turn improving vitamin D status of the breastfed infant (28). Unlike in many countries (49), vitamin D supplementation of breastfed infants is not a national policy recommendation in Bangladesh. Where maternal vitamin D deficiency and breastfeeding are both common, maternal supplementation during lactation can be an effective means of improving both maternal and infant vitamin D status (28). Our analysis shows only a weak association between vitamin D status at birth and later circulating 25(OH)D, underscoring early infancy as a period in which vitamin D supplementation may be particularly important.

Vitamin D–dependent gene transcription has long been recognized to involve heterodimerization of the vitamin D receptor with retinoid X receptor in the cell nucleus (50), yet the combined effect of vitamin D and vitamin A status on health outcomes is uncertain (51–53); in the present study, serum retinol was not associated with 25(OH)D. The potential reciprocal relation between folate and 25(OH)D was of particular interest given prior evidence that folate might contribute to the epigenetic regulation of vitamin D metabolism; through reduced methylation of the vitamin D Cytochrome P450 24-hydroxylase (*CYP24A1*), folate could promote a higher or sustained 25(OH)D concentration as the rate of catabolism declines (54). Placenta-specific methylation might be especially important for determining maternal-fetal transfer and, hence, neonatal 25(OH)D concentrations (55). Despite attenuation in multivariable models, maternal serum folate was positively associated with attained 25(OH)D at delivery, consistent with previous observations that folic acid supplementation may increase 25(OH)D (56). Conversely, the negative association of maternal folate with neonatal 25(OH)D did not support the hypothesis that folate regulates transplacental 25(OH)D transfer. Although we did not have available data to explore the influence of postnatal folate status on later 25(OH)D, our findings nonetheless highlight the potential confounding effect of multiple micronutrient supplementation on biomarker responses when compared with single nutrients in isolation (36).

Between-laboratory differences, and hence variable quality of 25(OH)D data, have long been raised as concerns for the reporting of 25(OH)D and other biomarker data (25, 57). In the present study, we have shown that despite low inter- and intra-assay CVs, assay drift and the time from supplement consumption to blood draw were associated with 25(OH)D. However, the effects were inconsistent, might have been partly confounded by other factors included in the models (e.g., season), and did not contribute substantially to total variance.

The well-characterized population and dose-ranging design are particular strengths of this study, permitting exploration of several potential contributors to interindividual variability in 25(OH)D across a range of administered doses. Unlike previous trials (10, 58), the relatively high prevalence of vitamin D deficiency in our population at enrollment enabled examination of this variability in a population in which intervention is likely to be of most benefit. We acknowledge the reduction in sample size in the present analysis compared with the parent trial as a limitation, which also contributed to inconsistent sample sizes across the statistical models. Given the diverse range of factors and exploratory nature of this analysis, we chose not to apply multiple imputation methods to address missingness. However, the detailed description of sample sizes for all completed analyses, and the similarity of participant characteristics across each time point, support our assumption that the findings are unlikely to have been affected by selection biases. We lacked measurements of other vitamin D metabolites, including circulating 24,25-dihydroxyvitamin D or vitamin D-3 itself, which might provide additional insights. Although the extent to which epigenetic regulation influences vitamin D metabolism and function remains unclear, common polymorphisms have been associated with the response to vitamin D supplementation during pregnancy (40), and might partially explain interindividual variations not captured by supplementation dose or basal vitamin D status. Initial 25(OH)D accounted for vitamin D inputs from dietary sources and cutaneous production before the start of each interval, but data were unavailable to quantify the effects of subsequent changes in maternal diet or sun exposure during the interval. Given the absence of vitamin D–rich or fortified foods in Bangladesh (22), we expected variation in 25(OH)D due to dietary sources to be minor in this study setting.

These findings support the expectation that the biochemical response of most pregnant women to a given dose of supplemental vitamin D can be reliably predicted, assuming good adherence. Given substantial between-population variation in average 25(OH)D during pregnancy (1), mean maternal vitamin D status of a target population should be considered in the determination of the recommended vitamin D dose (5, 58). Although higher BMI attenuated the 25(OH)D response at the highest vitamin D dose level, BMI did not contribute substantially to the overall variance in the 25(OH)D response after accounting for baseline vitamin D status. However, further research in populations with a higher prevalence of overweight/obesity is required to assess the potential advantages of customization of prenatal or postpartum vitamin D dosing based on BMI.

## Supplementary Material

nxab265_Supplemental_File

## Data Availability

Data described in the manuscript, code book, and analytic code will be made available upon request to the authors. Deidentified individual participant data will be provided for use in secondary data analyses approved by an independent research ethics board, and data requesters will be required to sign a data access agreement.

## Notes

This work was supported, in whole or in part, by the Bill & Melinda Gates Foundation [Grant Number OPP1066764]. Under the grant conditions of the Foundation, a Creative Commons Attribution 4.0 Generic License has already been assigned to the Author Accepted Manuscript version that might arise from this submission The funding agency was not involved in the design, implementation, analysis, or interpretation of the data.

Author disclosures: The authors report no conflicts of interest.

Supplemental Tables 1–5 and Supplemental Figures 1–3 are available from the “Supplementary data” link in the online posting of the article and from the same link in the online table of contents at https://academic.oup.com/jn/.

Abbreviations used: CRP, C-reactive protein; DEQAS, Vitamin D External Quality Assessment Scheme; DRV, Dietary Reference Value; LC-MS/MS, liquid chromatography–tandem mass spectrometry; LLoQ, lower limit of quantification; 25(OH)D, 25-hydroxyvitamin D; 25(OH)D_2_, 25-hydroxyvitamin D_2_; 25(OH)D_3_, 25-hydroxyvitamin D_3_.
