# Supplementary material for: Basal Vitamin D Status and Supplement Dose Are Primary Contributors to Maternal 25-Hydroxyvitamin D Response to Prenatal and Postpartum Cholecalciferol Supplementation
Source: J Nutr. 2021 Jul 24;151(11):3361–78. doi: 10.1093/jn/nxab265 (PMC8562081; doi:10.1093/jn/nxab265)
Supplement: nxab265_Supplemental_File [file nxab265_supplemental_file.pdf]

Basal vitamin D status and supplement dose are primary contributors to maternal 25-hydroxyvitamin D response to prenatal and postpartum cholecalciferol supplementation

Benjamin Levy, Karen M. O'Callaghan, Huma Qamar, Abdullah Al Mahmud, Alison D Gernand, M Munirul Islam, Daniel E. Roth.

Online supplementary material

Address correspondence to: Daniel E. Roth, Department of Paediatrics, Hospital for Sick Children and University of Toronto, 686 Bay Street, Toronto, ON, M5G 0A4, Canada; daniel.roth@sickkids.ca

25(OH)D response to vitamin D in pregnancy
Supplementary appendix
July 12, 2021

Basal vitamin D status and supplement dose are primary contributors to maternal 25-hydroxyvitamin D response to prenatal and postpartum cholecalciferol supplementation

Benjamin Levy et al.

Online Supplementary Material

Table of Contents

Supplemental Table 1: Assay information and performance indicators for biochemical measurements.. 2

Supplemental Table 2: Hypothesized predictors included in analyses of the 25-hydroxyvitamin D response to vitamin D supplementation, at each time point. 3

Supplemental Table 3: Sample sizes for hypothesized predictors of attained serum 25-hydroxyvitamin D concentrations in unadjusted and adjusted models, by time point. 5

Supplemental Table 4: Number of missing covariates and final sample sizes for multivariable models of attained serum 25-hydroxyvitamin D concentrations, by time point............................ 7

Supplemental Table 5: Comparison of model fit and variance explained (R^2) for multivariable models of attained serum 25-hydroxyvitamin D concentrations, by time point……………………..8

Supplemental Figure 1: Residual versus fitted plots showing the relationship between umbilical cord and maternal delivery 25(OH)D concentrations... 9

Supplemental Figure 2: Predictive margins for attained maternal serum 25(OH)D at delivery at a given supplemental vitamin D intake, stratified by vitamin D status at enrollment................. 10

Supplemental Figure 3: (A) Difference in maternal 25(OH)D concentrations at delivery in response to a 1-unit increase in each continuous predictor variable, adjusting for weekly prenatal vitamin D intake and serum 25(OH)D concentration at enrollment. (B) Change in maternal serum 25(OH)D concentrations at delivery compared to the reference group of each categorical predictor variable, adjusting for weekly prenatal vitamin D intake and serum 25(OH)D concentration at enrollment... 11

Supplemental Table 1: Assay information and performance indicators for biochemical measurements.

**Biochemical measure**	**Maternal or infant measurement**	**Biological matrix**	**Laboratory (institution)**	**Method**	**Assay kit/platform (manufacturer)**	**LLoQ**	**Imputation value if <LLoQ[1]**	**Samples <LLoQ *n* (%)**		**Inter-assay CV**	**Intra-assay CV**	**External proficiency testing/ QC material (program)**
								**Maternal**	**Cord/Infant**			
25(OH)D_3	Maternal & infant	Serum	AFBM (SickKids)	LC-MS/MS	Agilent 1290 HPLC system (Agilent Technologies); Q-TRAP 5500 mass spectrometer (Sciex)	1.25 nmol/L	0.624 nmol/L	0	1 (0.3)	7.0 %	4.9 %	NIST SRM972a QC material; External standards 451, 452, 453, 454 & 455 (DEQAS)
Ferritin	Maternal	Serum	NBL (icddr,b)	ECLIA	Elecsys Gen.2 Kit on Cobas e601 (Roche Diagnostics)	0.50 ng/mL	NR	N/A		3.2 %	-	
	Infant	Serum	CBL (icddr,b)	CMIA	ARCHITECT Kit # 7K59 (Abbott Laboratories)	<1 ng/mL	1 ng/mL		1 (0.1)	-	-	
CRP[2]	Maternal & infant	Plasma	SickKids	ELISA	Quantikine Kit # DCRP00 (R&D Systems)	0.78 µg/mL	0.39 µg/mL	11 (1.2)	90 (35)	7.4 %	5.1 %	
Folate	Maternal	Serum	NBL (icddr,b)	ECLIA	Roche Diagnostics		NR	N/A		4.2 %	-	
Retinol	Maternal	Serum	NBL (icddr,b)	RP-HPLC	Shimadzu Corporation		NR	N/A				NIST SRM968e QC material (VITAL-EQA)
Creatinine	Infant	Serum	CBL (icddr,b)	Colorimetry	Cayman Chemical		NR	N/A		-	-	

[1]NR denoted for biomarkers for which all measured concentrations were above the quantification limit. 25(OH)D_3, 25-hydroxyvitamin D_3; AFBM, Analytical Facility for Bioactive Molecules; CBL, Clinical Biochemistry Laboratory; CMIA, chemiluminescent microparticle immunoassay; CRP, C-reactive protein; DEQAS, Vitamin D External Quality Assessment Scheme; ECLIA, electrochemiluminescence immunoassay; ELISA, enzyme-linked immunoassay; icddr,b, International Centre for Diarrhoeal Disease Research, Bangladesh; LC-MS/MS, liquid chromatography-tandem mass spectrometry; LLoQ; lower limit of quantification; NBL, N/A, not applicable; Nutritional Biochemistry Laboratory at icddr,b; NIST, National Institute of Standards and Technology; NR, not required; RP-HPLC, reverse-phase high-performance liquid chromatography; SickKids, Hospital for Sick Children, Toronto, Canada; VITAL-EQA, Vitamin A Laboratory – External Quality Assurance program.
[2]Samples diluted 1000-fold.

Supplemental Table 2: Hypothesized predictors included in analyses of the 25-hydroxyvitamin D response to vitamin D supplementation, at each time point[1].

Variable	Unit	Maternal delivery	Maternal 6 months postpartum	Umbilical cord	Infant 6 months of age
Calculated vitamin D dose/week	IU/week	✓	✓	✓	✓
Initial serum 25(OH)D	nmol/L	✓	✓	✓	✓
Maternal characteristics					
Maternal age at enrollment	Years	✓	✓	✓	✓
Maternal height at enrollment	cm	✓	✓	✓	✓
Maternal BMI at enrollment	kg/m^2	✓		✓	
Maternal habitual BMI[2]	kg/m^2		✓	✓	
Parity[3]	-	✓	✓	✓	✓
Maternal education level	-	✓	✓	✓	✓
Asset index[4]	-	✓	✓	✓	✓
Maternal *paan* use[5]	-	✓	✓		
Maternal calcium intake[6]	mg/d	✓	✓	✓	
Maternal plasma CRP at enrollment	μg/mL	✓		✓	
Maternal serum retinol at delivery	μg/dL	✓		✓	
Maternal serum ferritin at delivery	ng/mL	✓		✓	
Maternal serum folate at delivery	ng/mL	✓		✓	
Placental weight	g			✓	
Infant characteristics					
Gestational age at birth	Weeks			✓	✓
Birth weight	g			✓	✓
Sex	-				✓
Infant serum creatinine at 6 months	ng/mL				✓
Infant serum CRP at 6 months	μg/mL				✓
Breastfeeding pattern[7]	-				✓
Specimen- and Laboratory-related factors					
Duration of supplementation	Weeks	✓	✓	✓	
Season of blood collection	-	✓	✓	✓	✓
Time of day of blood collection[8]	-	✓	✓	✓	✓
Time between blood collection and 25(OH)D analysis[9]	Months	✓	✓	✓	✓
Dose-to-sampling timing[10]	Days	✓	✓	✓	
Assay drift[11]	Months	✓	✓	✓	✓

[1]Includes all variables considered to influence the vitamin D intake-25-hydroxyvitamin D response relationship. 25(OH)D, 25-hydroxyvitamin D; CRP, C-reactive protein.
[2]BMI at 12 months postpartum was used as a proxy for habitual BMI in the non-pregnant, non-lactating state.
[3]Defined based on the total number of previous live births, irrespective of previous miscarriage or abortions, and was inclusive of the current pregnancy. Hence, parity was categorized as primiparous (no previous live birth) or multiparous (≥1 live birth).
[4]Determined by ownership of household items, using principal components analysis.
[5]Locally-defined term referring to a preparation of areca nut wrapped in betel leaf, that is chewed in a manner similar to tobacco.
[6]Estimated by means a targeted, non-quantitative food frequency questionnaire.
[7]Defined as exclusive/predominant breastfeeding (breast milk in addition to water, sugar water, honey, or other non-milk, non-formula liquid), partial breastfeeding (breast milk with animal, powdered or condensed milk, and solid or semi-solid foods) or formula feeding at 6 months of age.
[8]Categorized as morning (00:00 to 11:59 hrs), afternoon (12:00 to 16:59 hrs), or evening (17:00 to 23:59 hrs).
[9]Expressed in months and used to examine potential minor degradation during freezer storage.
[10]Defined as the number of days between blood sample collection and the last administered vitamin D (or placebo) intervention dose.
[11]Estimated by the number of months between completion of the first 25(OH)D assay and the assay concerning the 25(OH)D measurement of interest.

Supplemental Table 3: Sample sizes for hypothesized predictors of attained serum 25-hydroxyvitamin D concentrations in unadjusted and adjusted models, by time point[1].

	Maternal delivery		Maternal 6 months postpartum		Umbilical cord		Infant 6 months of age	
	Unadjusted	Adjusted	Unadjusted	Adjusted	Unadjusted	Adjusted	Unadjusted	Adjusted
**Serum 25(OH)D concentration**	**655**	**651**	**566**	**566**	**502**	**500**	**215**	**215**
Maternal age	655	651	566	566	502	500	215	215
Maternal education level	655	651	566	566	502	500	215	215
Asset index[2]	653	649	564	564	500	498	215	215
Parity[3]	655	651	566	566	502	500	215	215
Days between last dose and blood collection	655	651	566	566	500	500	212	212
Months since first assay (assay drift)	655	651	566	566	502	500	215	215
Months between blood collection and assay	655	651	566	566	502	500	215	215
Time of day of blood collection	655	651	566	566	502	500	215	215
Number of weeks of supplementation	655	651	566	566	502	500	215	215
Maternal height	655	651	566	566	502	500	215	215
Maternal BMI at enrollment	630	626	-	-	483	481	-	-
Maternal habitual BMI[4]	-	-	553	553	-	-	210	210
Maternal delivery serum folate	648	644	-	-	494	494	-	-
Maternal delivery serum retinol	496	493	-	-	404	404	-	-
Maternal delivery serum ferritin	648	644	-	-	494	494	-	-
Maternal enrollment plasma CRP	607	604	-	-	498	496	-	-
Maternal prenatal daily calcium intake[5]	655	651	-	-	502	500	-	-
Maternal postpartum daily calcium intake[5]	-	-	562	562	-	-	-	-
Season of delivery	653	649	-	-	507	500	-	-
Season at 6 months postpartum	-	-	566	566	-	-	215	215
Placental weight	-	-	-	-	496	494	-	-
Gestational age at birth	-	-	-	-	502	500	-	-
Infant birth weight	-	-	-	-	438	437	186	186
Infant 6 month serum creatinine	-	-	-	-	-	-	210	210
Infant 6 month serum ferritin	-	-	-	-	-	-	210	210
Infant 6 month plasma CRP	-	-	-	-	-	-	133	133
Infant sex	-	-	-	-	-	-	215	215
Breastfeeding pattern[6]	-	-	560	560	-	-	212	212

[1]Sample size refers to participants for whom both the predictor variable and serum 25-hydroxyvitamin D (25(OH)D) is available at the time point of interest. Unadjusted model includes the listed variable as a single predictor variable of 25(OH)D only, whereas adjusted model includes the listed variable with adjustment for vitamin D intake and initial (start-of-interval) 25(OH)D concentration. 25(OH)D, 25-hydroxyvitamin D; CRP, C-reactive protein.

[2]Determined by ownership of household items, using principal components analysis.
[3]Defined based on the total number of previous live births, irrespective of previous miscarriage or abortions, and was inclusive of the current pregnancy. Hence, parity was categorized as primiparous (no previous live birth) or multiparous (≥1 live birth).
[4]BMI at 12 months postpartum was used as a proxy for habitual BMI in the non-pregnant, non-lactating state.
[5]Estimated by means a targeted, non-quantitative food frequency questionnaire.
[6]Defined as exclusive/predominant breastfeeding (breast milk in addition to water, sugar water, honey, or other non-milk, non-formula liquid), partial breastfeeding (breast milk with animal, powdered or condensed milk, and solid or semi-solid foods) or formula feeding at 6 months of age.

Supplemental Table 4: Number of missing covariates and final sample sizes for multivariable models of attained serum 25-hydroxyvitamin D concentrations, by time point.

	Maternal delivery	Maternal 6 month postpartum	Umbilical cord	Infant 6 months of age
**Serum 25(OH)D concentration**	**655**	**566**	**502**	**215**
Missing data[1]				
Initial 25(OH)D concentration[2]	4	0	2	0
Maternal enrollment BMI	25	-	19	-
Asset index[3]	2	2	2	0
Maternal enrollment plasma CRP	44	-	4	-
Maternal delivery serum folate	6	-	6	-
Maternal delivery serum retinol	103	-	83	-
Placental weight	-	-	4	-
Infant birth weight	-	-	53	29
Maternal habitual BMI[4]	-	13	-	5
Postpartum daily calcium intake[5]	-	4	-	-
Breastfeeding pattern[6]	-	6	-	3
Infant 6 month serum ferritin	-	-	-	4
**Included in multivariable models**	**471**	**541**	**329**	**174**

[1]The value in each row represents the number of participants excluded from analysis due to missingness of the listed covariate, if not already accounted for by missingness of the preceding covariate. Only covariates that contributed to missing data (thereby reducing the final sample size of the multivariable model) are listed: 0 represents the variables that were included in the multivariable model at a given time point and for which all data was available (i.e., 0 data points missing); the hyphen represents the variables not included in the multivariable model at that time point. Covariates are listed in order from highest to lowest importance in terms of expected contribution to 25(OH)D response, hypothesized *a priori*. 25(OH)D, 25-hydroxyvitamin D; CRP, C-reactive protein.
[2]Defined as the 25(OH)D measurement at the preceding time point.
[3]Determined by ownership of household items, using principal components analysis
[4]BMI at 12 months postpartum was used as a proxy for BMI in the non-pregnant, non-lactating state.
[5]Estimated by a targeted, non-quantitative food frequency questionnaire.
[6]Defined as exclusive/predominant breastfeeding (breast milk in addition to water, sugar water, honey, or other non-milk, non-formula liquid), partial breastfeeding (breast milk with animal, powdered or condensed milk, and solid or semi-solid foods) or formula feeding at 6 months of age.

Supplemental Table 5: Comparison of model fit and variance explained (R^2) for multivariable models of attained serum 25-hydroxyvitamin D concentrations, by time point[1].

	R^2	AIC	P(F>f)
**Maternal delivery**			
Base model[2]	0.70	4254.9	<0.001
Multivariable model C[3]	0.73	**4228.8**	**<0.001**
Multivariable model D[4]	0.75	4242.6	0.17
**Maternal 6 months postpartum**			
Base model[2]	0.79	4436.6	<0.001
Multivariable model C[3]	0.82	**4379.5**	**<0.001**
Multivariable model D[4]	0.82	4388.7	0.45
**Umbilical cord**			
Base model[2]	0.82	2502.4	<0.001
Multivariable model C[3]	0.85	**2500.2**	**<0.001**
Multivariable model D[4]	0.85	2512.7	0.50
**Infant at 6 months of age**			
Base model[2]	0.24	1595.1	<0.001
Multivariable model C[3]	0.38	**1581.5**	**<0.001**
Multivariable model D[4]	0.43	1581.9	0.13

[1]For each time point, the best-fit model based on both partial F-test and lowest AIC value is highlighted in bold. *P*-value for partial F-tests were determined based on the comparison of two regression models in a nested sequence and test the null hypothesis that the sum of squared residuals following addition of variables to each model did not change. The base model was compared to a model with one intercept term only; multivariable model C was compared to the base model; and multivariable model D was compared to multivariable model C.
[2]Base model included only initial 25-hydroxyvitamin D (25(OH)D) and calculated vitamin D dose/week as predictors of attained 25(OH)D. For the maternal delivery and 6-month postpartum time points, initial 25(OH)D was defined as enrollment and delivery 25(OH)D concentrations, respectively. For neonatal and infant 6-month 25(OH)D, initial 25(OH)D was defined as maternal delivery and cord 25(OH)D, respectively.
[3]Model C was a parsimonious multivariable model including initial 25(OH)D, calculated vitamin D dose/week, any predictor variables with a *P*-value of <0.1 in in either a bivariate (unadjusted) model with 25(OH)D as the dependent variable or in an extended model adjusting for initial 25(OH)D and calculated vitamin D dose/week, as well as variables hypothesized *a priori* to be a determinant of attained 25(OH)D (season of blood draw, maternal enrollment or habitual BMI (measured at 12 months postpartum), and, for infants, breastfeeding pattern). Interaction terms between predictor variables and supplemental vitamin D intake were included where applicable (see Tables 2-5 in main manuscript).
[4]Model D included all variables of interest (see Supplemental Table 2). Interaction terms between predictor variables and supplemental vitamin D intake were included where applicable (see Tables 2-5 in main manuscript).

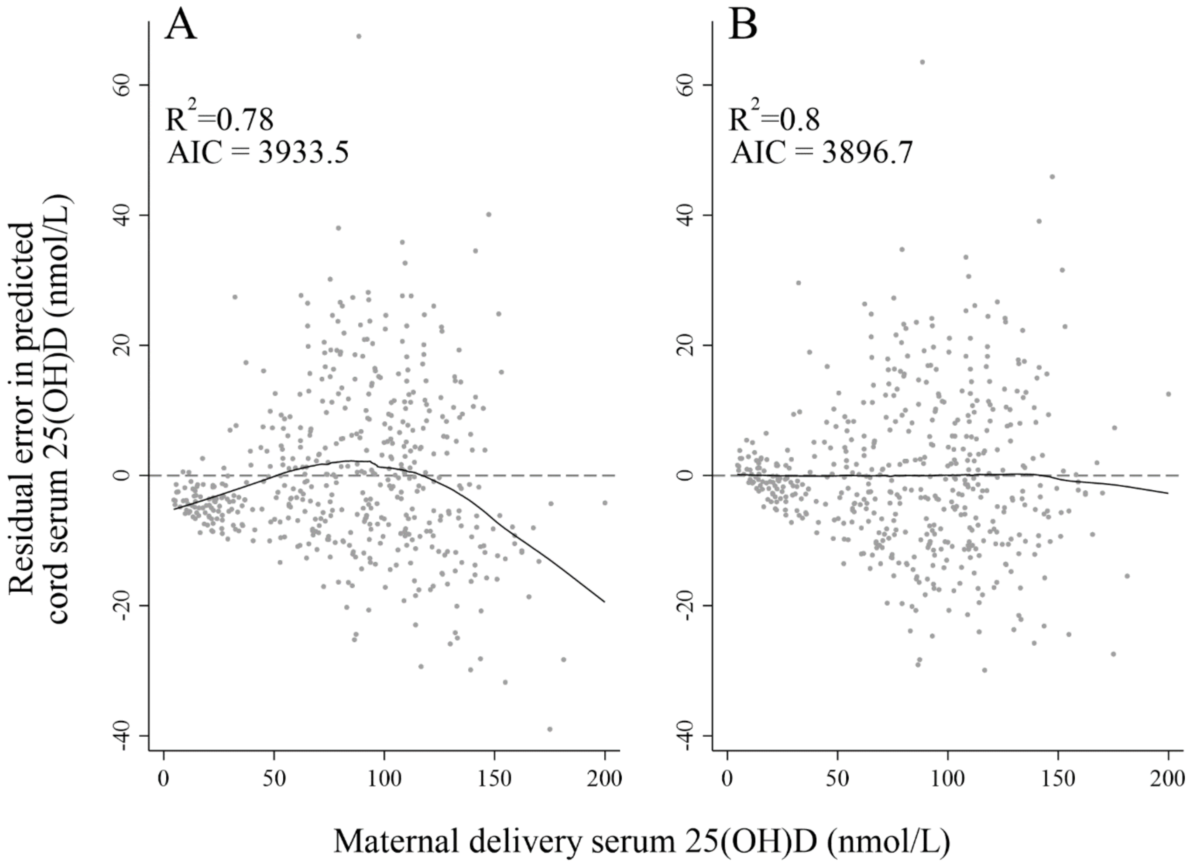

Supplemental Figure 1: Residual versus fitted plots showing the relationship between umbilical cord serum and maternal delivery 25(OH)D concentrations. (A) Residuals from a linear model showed clear nonlinearity; (B) A restricted cubic spline model, with knots at mean maternal 25(OH)D at delivery for each treatment group, shows no discernible pattern between residuals and maternal delivery 25(OH)D. The black lines represent LOWESS curves fitted through the residuals. Maternal delivery and umbilical cord 25(OH)D concentrations were highly correlated (*n*=500; Spearman ρ=0.87, *P*<0.001). 25(OH)D, 25-hydroxyvitamin D; AIC, Akaike's information criterion.

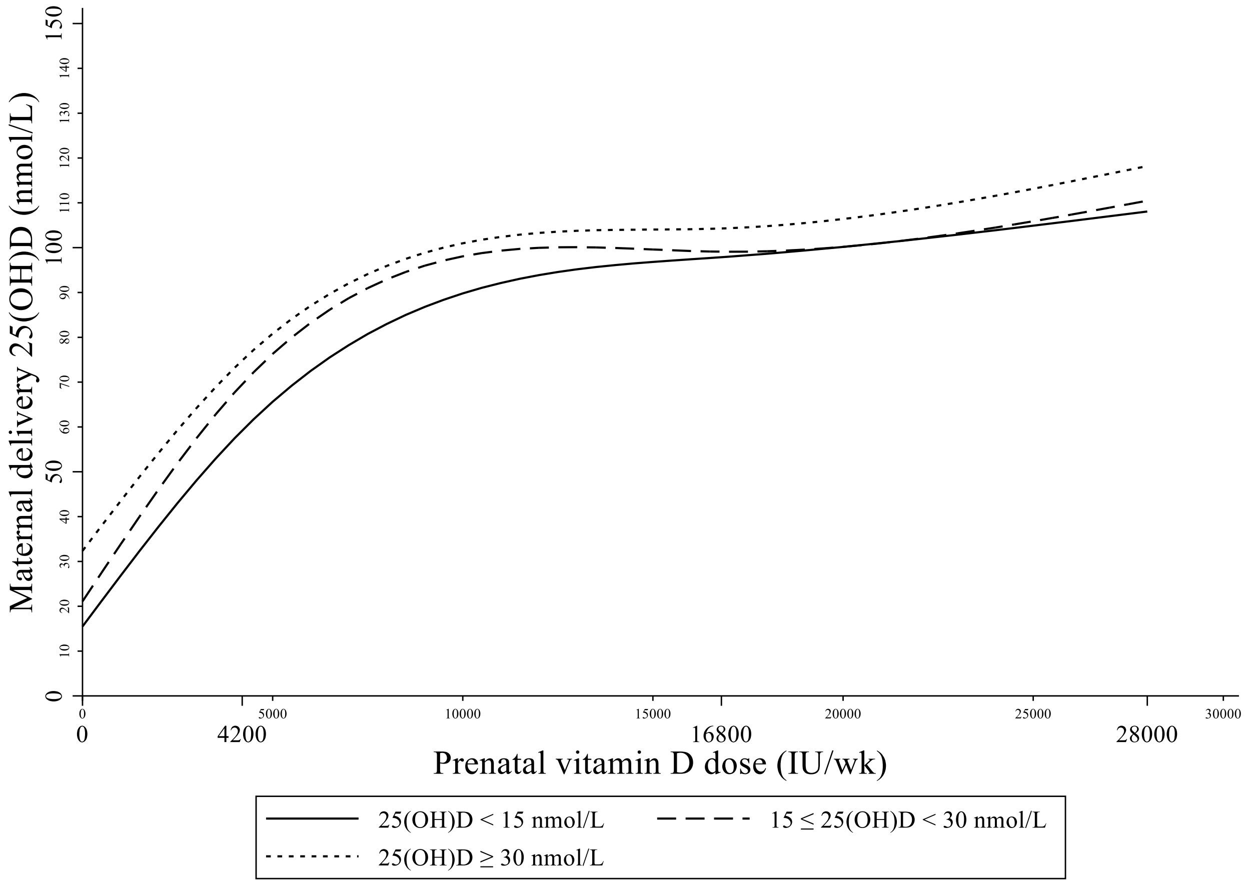

Supplemental Figure 2: Predictive margins for attained maternal serum 25(OH)D at delivery at a given supplemental vitamin D intake, stratified by categories of vitamin D status at enrollment. Linear regression models showed a non-significant interaction between categorized vitamin D status at enrollment and supplemental vitamin D intake (*P*=0.71), whereby statistical significance was determined from the F-test joint assessment of significance across each level of vitamin D status. 25(OH)D, 25-hydroxyvitamin D. Among women randomized to placebo, mean 25(OH)D at delivery was 11.3 nmol/L higher (95% CI: 2.6, 19.9; *P*=0.011) in women who were relatively replete (≥30 nmol/L) at enrollment compared to women with moderate deficiency (≥15 nmol/L 25(OH)D <30 nmol/L); differences were slightly attenuated when comparing vitamin D replete versus moderately-deficient women among those who received supplemental vitamin D: 5.3 nmol/L (95% CI: -3.6, 14.1; *P*=0.24), 5.2 nmol/L (95% CI: -2.9, 13.3; *P*=0.21) and 7.7 nmol/L (95% CI: 1.6, 13.8; *P*=0.013) in the 4200 IU/week, 16800 IU/week and 28000 IU/week groups, respectively. Mean attained 25(OH)D following prenatal supplementation was lowest among women with very poor (<15 nmol/L) vitamin D status relative to women with moderate deficiency, but this was only statistically significant in the 4200 IU/week trial arm (-10.4 nmol/L; 95% CI: -20.4, -0.33; *P*=0.043).

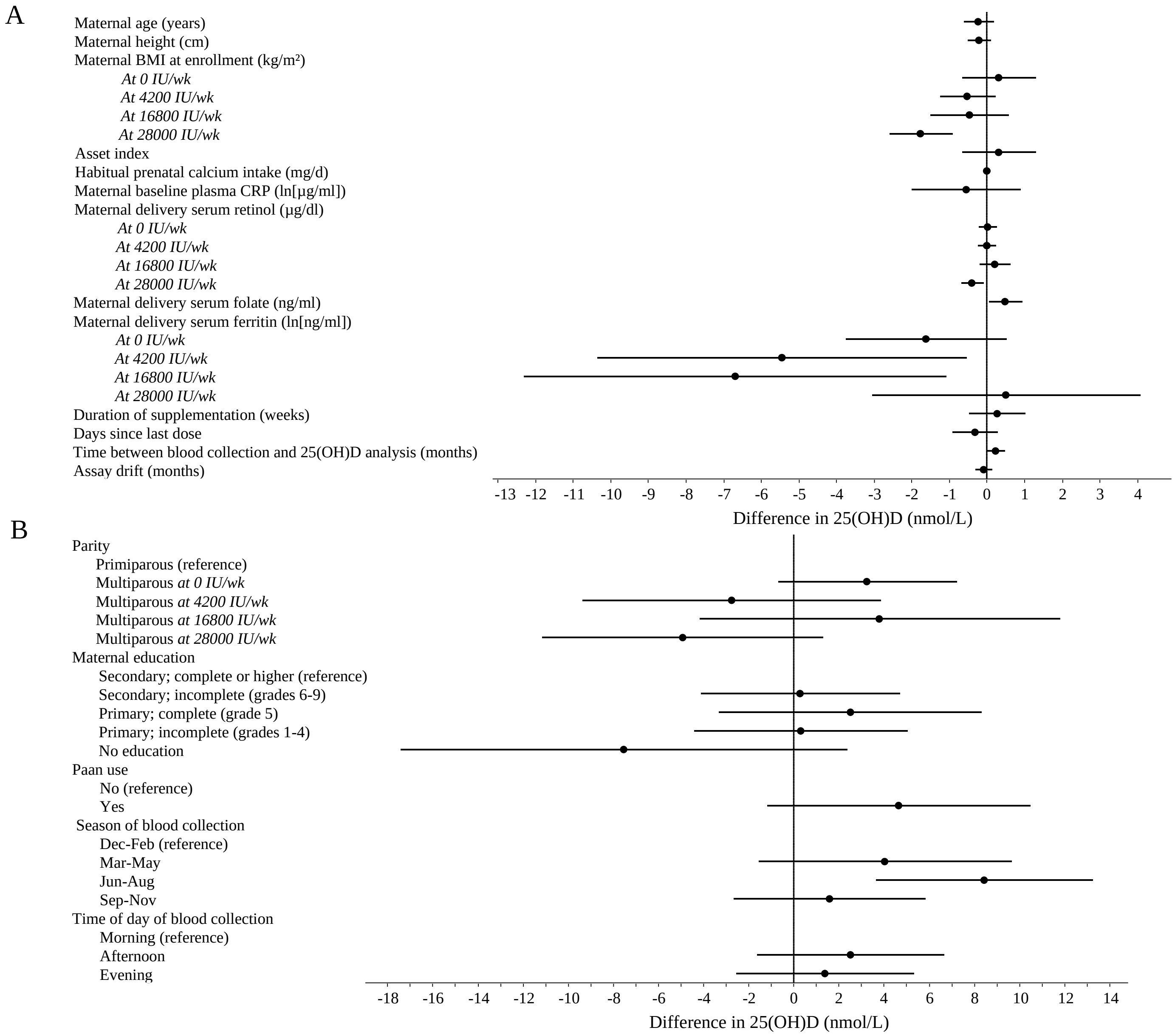

Supplemental Figure 3: (A) Difference in maternal 25(OH)D concentrations at delivery in response to a 1-unit increase in each continuous predictor variable, adjusting for weekly prenatal vitamin D intake and serum 25(OH)D concentration at enrollment. (B) Change in maternal serum 25(OH)D concentrations at delivery compared to the reference group of each categorical predictor variable, adjusting for weekly prenatal vitamin D intake and serum 25(OH)D concentration at enrollment. One individual model was created for each listed predictor (Model B). Statistical interactions between vitamin D intake and maternal enrollment BMI, delivery ferritin, and delivery retinol were expressed as average marginal effects, fixing vitamin D intake at each of the assigned weekly prenatal dose provided (0, 4200, 16800, and 28000 IU/week); coefficients are predicted margins that represent the change in 25(OH)D for a one-unit increase in the predictor (continuous variable) or difference compared to the reference category (categorical variable), at the specified vitamin D dose level. Mean difference in maternal delivery 25(OH)D and 95% CI for each treatment group is represented by the solid black circles and horizontal lines. *n*=651 for all predictors except: enrollment BMI (*n*=626); asset index (*n*=649); baseline CRP (*n*=604); delivery retinol (*n*=493); delivery folate (*n*=644); delivery ferritin (*n*=644) and; season of delivery (*n*=649). 25-hydroxyvitamin D, 25(OH)D; CRP, C-reactive protein.
